# Comparative genomics analysis of c-di-GMP metabolism and regulation in *Microcystis aeruginosa*

**DOI:** 10.1186/s12864-020-6591-3

**Published:** 2020-03-09

**Authors:** Meng Chen, Chun-Yang Xu, Xu Wang, Chong-Yang Ren, Jiao Ding, Li Li

**Affiliations:** 1 Shandong Provincial Key Laboratory of Water Pollution Control and Resource Reuse, School of Environmental Science and Engineering, Shandong University, Qingdao, China; 2 Shandong Provincial Engineering Center on Environmental Science and Technology, Jinan, China

**Keywords:** *Microcystis aeruginosa*, Comparative genomics, C-di-GMP, Phylogenetic analysis, GGDEF, EAL, HD-GYP, PilZ

## Abstract

**Background:**

Cyanobacteria are of special concern because they proliferate in eutrophic water bodies worldwide and affect water quality. As an ancient photosynthetic microorganism, cyanobacteria can survive in ecologically diverse habitats because of their capacity to rapidly respond to environmental changes through a web of complex signaling networks, including using second messengers to regulate physiology or metabolism. A ubiquitous second messenger, bis-(3′,5′)-cyclic-dimeric-guanosine monophosphate (c-di-GMP), has been found to regulate essential behaviors in a few cyanobacteria but not *Microcystis*, which are the most dominant species in cyanobacterial blooms. In this study, comparative genomics analysis was performed to explore the genomic basis of c-di-GMP signaling in *Microcystis aeruginosa*.

**Results:**

Proteins involved in c-di-GMP metabolism and regulation, such as diguanylate cyclases, phosphodiesterases, and PilZ-containing proteins, were encoded in *M. aeruginosa* genomes. However, the number of identified protein domains involved in c-di-GMP signaling was not proportional to the size of *M. aeruginosa* genomes (4.97 Mb in average). Pan-genome analysis showed that genes involved in c-di-GMP metabolism and regulation are conservative in *M. aeruginosa* strains*.* Phylogenetic analysis showed good congruence between the two types of phylogenetic trees based on 31 highly conserved protein-coding genes and sensor domain-coding genes. Propensity for gene loss analysis revealed that most of genes involved in c-di-GMP signaling are stable in *M. aeruginosa* strains. Moreover, bioinformatics and structure analysis of c-di-GMP signal-related GGDEF and EAL domains revealed that they all possess essential conserved amino acid residues that bind the substrate. In addition, it was also found that all selected *M. aeruginosa* genomes encode PilZ domain containing proteins.

**Conclusions:**

Comparative genomics analysis of c-di-GMP metabolism and regulation in *M. aeruginosa* strains helped elucidating the genetic basis of c-di-GMP signaling pathways in *M. aeruginosa*. Knowledge of c-di-GMP metabolism and relevant signal regulatory processes in cyanobacteria can enhance our understanding of their adaptability to various environments and bloom-forming mechanism.

## Background

Cyanobacteria, which are phototrophic bacteria that survive in ecologically diverse habitats, have received growing attention because they have been forming toxic blooms in eutrophic water bodies worldwide for decades [1, 2]. Dense blooms are considered seriously harmful to aquatic ecosystems because of their deleterious effects on water quality, such as increased turbidity, smothering submerged aquatic vegetation, and producing taste and odor compounds [3, 4]. Moreover, some cyanobacteria species can synthesize toxic secondary metabolites, such as hepatotoxin microcystins that can inhibit eukaryotic protein phosphatases; thus, they threaten the function of water bodies for drinking, bathing, and fishing, and they also ultimately pose potential risks to animal and human health [5–7]. Cyanobacteria are able to inhabit most of Earth’s environments because they evolved mechanisms to monitor and rapidly adapt to environmental changes through a web of complex signaling networks, such as using second messengers to regulate physiology or metabolism [8].

Cyanobacteria must cope with variations in the external environment. They rely on signaling molecules to translate these changes into intracellular responses and mediate adaptation to ambient conditions. Once bacterial cells sense an external stimulus, such as light and temperature, the intracellular level of a second messenger rapidly changes to amplify the biological input signal to a downstream output effector and initiate physiological changes, including sugar metabolism, motility, and biofilm production [8, 9]. A ubiquitous second messenger, bis-(3′,5′)-cyclic-dimeric-guanosine monophosphate (c-di-GMP), which was first identified as an allosteric activator of cellulose synthase in *Gluconacetobacter xylinus* in 1987, plays an important role in regulating biofilm formation or dispersal in response to various environmental cues and cell–cell signals [10–14]. Studies have summarized that c-di-GMP regulates an astounding array of important processes in bacteria, including transcription, RNA turnover, protein synthesis, motility, virulence, and altering activities of proteins or protein complexes [15–17]. The intracellular level of c-di-GMP are modified by the rate of its synthesis and degradation in response to a variety of environmental stimuli, relying on the opposite enzymatic activity of diguanylate cyclases (DGCs) and c-di-GMP-specific phosphodiesterases (PDEs), respectively [12, 18]. DGC proteins contain a GGDEF domain that synthesizes one c-di-GMP molecule from two GTP molecules [19, 20]. PDE proteins contain an EAL or, less frequently, a HD-GYP domain, which breaks down c-di-GMP into the linear molecule 5′-phosphoguanylyl-(3′-5′)-guanosine (pGpG) or into two GMP molecules [21, 22]. Moreover, GGDEF and EAL domains can both be present in the same protein, forming “hybrid” proteins, even though they have opposing activities [23, 24]. In that case, only one of the two domains is catalytically active, and the other performs a regulatory function, or a third regulatory domain is present that may disjoin the activity of the GGDEF and EAL domains [23, 25, 26]. Ute Römling et al. list a census of all GGDEF, EAL, and HD-GYP domains in bacterial genomes [12, 27].

**Fig. 1:**
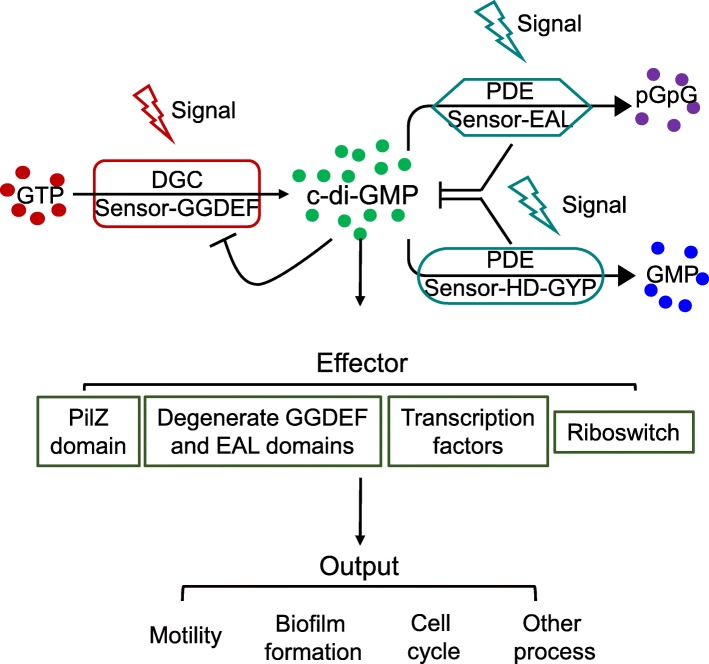
C-di-GMP metabolism and regulation module. Once bacterial cells sense environmental cues, such as light and temperature, the intracellular level of c-di-GMP rapidly changes to amplify the biological input signal. C-di-GMP then is recognized by downstream effector that interact with a downstream target to affect specific physiological processes. C-di-GMP synthesis and degradation is achieved by DGC proteins containing the GGDEF domain and PDE proteins bearing the EAL or HD-GYP domains, respectively. Downstream effectors contain PilZ, degenerate GGDEF or EAL domain, riboswitch, and transcription factors

Diverse sensor domains can modulate enzymatic activities in response to external stimuli, including N-terminal response regulator receiver (REC), Per/Arnt/Sim (PAS), histidine kinases/adenylate cyclases/methyl accepting proteins and phosphatases (HAMP), and cGMP phosphodiesterase/adenylyl cyclase/FhlA (GAF) domains, which were related to c-di-GMP association network retrieved by STRING [25, 28–30]. C-di-GMP has been found to be recognized by downstream receptors that have been linked to specific physiological processes, ranging from polysaccharide biosynthesis to direct regulation of gene expression and to motility. Among the downstream effectors, the PilZ domain is ubiquitous in bacteria and can bind c-di-GMP to regulate biosynthesis of biofilms, such as cellulose and alginate [31–33]. The PilZ domain can be a stand-alone protein or fused with other functional proteins, such as cellulose synthases and alginate biosynthesis protein, or attached to certain signaling domains, such as the GGDEF, EAL, and HD-GYP domains [32, 34] (Fig. 1). Molecular mechanisms of c-di-GMP signaling in a few cyanobacteria that are obligate photosynthetic microorganisms in the environment, such as *Thermosynechococcus* and *Synechocystis*, have been examined in-depth [35–38]. However, none of those studies have addressed *Microcystis*, one of the most ubiquitous freshwater cyanobacterial genera, which limits the comprehensive understanding of c-di-GMP signaling in cyanobacteria [36].

Genome sequencing of numerous *Microcystis* species has been performed, which makes it possible to improve our knowledge about c-di-GMP function in this genus. The purpose of this study was to explore the genomic basis of c-di-GMP signaling in *M. aeruginosa*. In this study, c-di-GMP metabolism and regulation in *M. aeruginosa* was revealed through in silico comparative analyses. We identified genes that encode proteins containing the GGDEF, EAL, HD-GYP and PilZ domains and other associated sensing domains in the complete or draft genome sequences of 24 *M. aeruginosa* strains available in GenBank. Meanwhile, we performed comparative genomic analyses based on phylogenetic, phylogenomic, positive selection, and pan-genome analyses of these strains to comprehensively analyze the c-di-GMP signaling genes. We also characterized the structural features of GGDEF, EAL, HD-GYP and PilZ domains. The comparative genomic analysis will help elucidate c-di-GMP metabolism and relevant signal regulation processes in cyanobacteria.

## Results

### General genome features of *M. aeruginosa* strains

Genomes of 24 *M. aeruginosa* strains were retrieved from the National Center for Biotechnology Information (NCBI) database for series analysis. As shown in Table 1, except for strains NIES 2481 [39] and NIES 2549 [40], no plasmid sequence was discovered in other strains. Among them, genome sequences of the strains CHAOHU 1326 and NaRes975 were recently released by our laboratory [41, 42], and their general information are shown in Table S1. The average size of the genomes was 4.97 ± 0.40 Mb, and the average G + C content was 42.66 ± 0.26%. Genomes ranged in size from 4.26 Mb (*M. aeruginosa* PCC9806) to 5.89 Mb (*M. aeruginosa* KW, Table 1). The selected genomes are of high completeness and low contamination as evaluated based on lineage-specific marker sets by checkM [43]. Multiple rRNA coding sequences were present in *M. aeruginosa* strains. Generally, each strain contains 1~2 sets of rRNA clusters as a rough estimation due to the incomplete sequences present in the genomes (Additional file 1, Table S2).

**Table 1:** Genome features of the 24 analyzed *M. aeruginosa* strains

Strains	Isolation Location	NCBI Accession Number (Genome/Plasmid)	NCBI Assembly Number	Contigs	Genome Size (Mbs)	G + C %	CDs	Completeness (%)	Contamination (%)
CHAOHU 1326	Chaohu Lake, CN	MOLZ00000000/−	GCA_001895325.1	617	5.27158	42.50	4590	99.67	1.39
DIANCHI905	Dianchi Lake, CN	AOCI00000000/−	GCA_000332585.1	335	4.85887	42.50	4303	99.01	2.92
KW	Wangsong Reservoir, KR	MVGR00000000/−	GCA_002025445.1	6	5.88943	42.80	4854	97.92	0.51
NaRes975	Nanwan Reservoir, CN	MOLN00000000/−	GCA_001885655.1	413	5.11753	42.40	4617	99.89	0.51
NIES44	Lake kasumigaura, JP	BBPA00000000/−	GCA_000787675.1	79	4.56532	43.20	4053	99.89	0.07
NIES87	Lake kasumigaura, JP	BFAC00000000/−	GCA_002933835.1	246	4.92578	42.90	4214	99.89	0.84
NIES88	Lake Kawaguchi Yamanashi, JP	JXYX00000000/−	GCA_001578075.1	262	5.26322	43.00	4620	99.45	0.84
NIES98	Lake kasumigaura, JP	MDZH00000000/−	GCA_001725075.1	500	4.98253	42.40	4412	99.67	0.37
NIES843	Lake kasumigaura, JP	AP009552/−	GCA_000010625.1	1	5.84279	42.30	5190	99.89	0.51
NIES1211	Lake Tofutsu, JP	BEIV00000000/−	GCA_003206625.1	289	4.73839	42.80	4209	99.89	0.51
NIES2481	Lake kasumigaura, JP	CP012375/CP025929	GCA_001704955.2	2	4.44055	42.86	3966	99.82	0.15
NIES2549	Lake kasumigaura, JP	CP011304/CP026286	GCA_000981785.2	2	4.3012	42.90	3843	99.89	0.07
PCC7806SL	Braakman Reservoir, NL	CP020771/−	GCA_002095975.1	1	5.13934	42.10	4497	99.67	1.45
PCC7941	Lake Lillte Rideau, CA	CAIK00000000/−	GCA_000312205.1	433	4.8019	42.60	4337	98.57	0.73
PCC9432	Lake Lillte Rideau, CA	CAIH00000000/−	GCA_000307995.2	438	4.99494	42.50	4543	99.67	0.29
PCC9443	Fishpond, CF	CAIJ00000000/−	GCA_000312185.1	760	5.18504	42.70	4545	98.36	0.37
PCC9701	Guerlesquin dam, FR	CAIQ00000000/−	GCA_000312285.1	550	4.756	42.70	4312	99.12	0.07
PCC9717	Rochereau dam, FR	CAII00000000/−	GCA_000312165.1	892	5.30034	42.70	4609	98.57	0.29
PCC9806	Oskosh, US	CAIL00000000/−	GCA_000312725.1	310	4.26256	43.10	4258	99.01	0.18
PCC9807	Hartbeespoort dam, ZA	CAIM00000000/−	GCA_000312225.1	782	5.15571	42.60	4588	99.01	0.91
PCC9808	Malpas dam, AU	CAIN00000000/−	GCA_000312245.1	479	5.05105	42.40	4556	99.01	0.51
PCC9809	Lake Michigan, US	CAIO00000000/−	GCA_000312265.1	809	5.01102	42.80	4497	98.36	0.95
Sj	Lake Shinji, JP	BDSG00000000/−	GCA_003206555.1	366	4.61732	42.80	3956	99.67	0.18
TAIHU98	Taihu Lake, CN	ANKQ00000000/−	GCA_000330925.1	4	4.84961	42.50	4340	99.89	0.22

### Modular signaling proteins involved in c-di-GMP metabolism and regulation in *M. aeruginosa*

A genome search for genes that encode enzymes involved in c-di-GMP metabolism was performed to identify the putative translated products that have DGC and PDE activities in the selected 24 *M. aeruginosa* genomes. The accession numbers of the predicted proteins are shown in Table 2. This survey led to identification of three enzymatic classes of predicted proteins DGCs, PDEs, and hybrid DGC–PDEs, which contain GGDEF and EAL domains, even though they have opposing activities. As listed in Tables 2, 14 of the 24 *M. aeruginosa* genomes had genes that encode DGC enzymes, which contain a fused N-terminal REC domain and GGDEF domain in tandem. The REC domain, as a signal receiver domain present in association with c-di-GMP metabolism domains, is supposed to modulate the enzymatic activities in response to the internal or external stresses. There are two types of PDEs in *M. aeruginosa* genomes, one type contains partial EAL domain, and the other type contains HD-GYP domain along with a N-terminal DICT domain, a sensory domain in “diguanylate cyclases and two-component system”. Interestingly, compared with the HD-GYP domain-containing PDEs, which were identified in all selected *M. aeruginosa* genomes and seemed to be highly conserved, proteins with partial EAL domains were found less frequently (in only three genomes). Except for the NIES44 genome, each of the other 23 genomes was found to have a GG [D/E]EF-EAL hybrid protein, consisting of GGDEF, EAL domain, and Forkhead associated (FHA) domain, a putative nuclear signaling domain.

**Table 2:** Predicted modular signaling proteins involved in c-di-GMP metabolism in all 24 analyzed *M. aeruginosa* genomes

Strains	DGC (REC-GGDEF)^a^	PDE (DICT-HD-GYP)	PDE (EAL)	Hybrid protein (FHA-GGDEF-EAL)	DGC, PDE, Hybrid protein^b^
CHAOHU 1326	WP_052276147.1	WP_052275339.1	–	WP_052277914.1	1, 1, 1
DIANCHI905	–	WP_002746813.1	–	WP_002743531.1	0, 1, 1
KW	WP_079210059.1	WP_002796380.1	–	WP_079210289.1	1, 1, 1
NaRes975	–	WP_002752229.1	–	WP_044034220.1	0, 1, 1
NIES44	–	WP_045358386.1	–	–	0, 1, 0
NIES87	–	WP_104396273.1	–	WP_104397223.1	0, 1, 1
NIES88	WP_061433230.1	WP_061432432.1	–	WP_061431785.1	1, 1, 1
NIES98	–	WP_002752229.1	–	WP_002739484.1	0, 1, 1
NIES843	WP_012264732.1	WP_002796380.1	–	WP_012266621.1	1, 1, 1
NIES1211	WP_039900524.1	WP_039900517.1	WP_071989022.1	WP_110544382.1	1, 1, 1
NIES2481	WP_046660716.1	WP_066029831.1	WP_080949698.1	WP_066029445.1	1, 1, 1
NIES2549	WP_046660716.1	WP_046662116.1	WP_080949698.1	WP_046660636.1	1, 1, 1
PCC7806SL	–	WP_002746813.1	–	WP_002743531.1	0, 1, 1
PCC7941	–	WP_002752229.1	–	WP_043997363.1	0, 1, 1
PCC9432	–	WP_002752229.1	–	WP_002750015.1	0, 1, 1
PCC9443	WP_043996837.1	WP_002765696.1	–	WP_002768060.1	1, 1, 1
PCC9701	WP_002801860.1	WP_002803155.1	–	WP_004163835.1	1, 1, 1
PCC9717	WP_043999403.1	WP_002762031.1	–	WP_002761714.1	1, 1, 1
PCC9806	WP_002783698.1	WP_002780038.1	–	WP_002783280.1	1, 1, 1
PCC9807	WP_002787322.1	WP_002785975.1	–	WP_004161732.1	1, 1, 1
PCC9808	–	WP_002752229.1	–	WP_044034220.1	0, 1, 1
PCC9809	WP_043999403.1	WP_002796380.1	–	WP_002797049.1	1, 1, 1
Sj	WP_110579156.1	WP_110579081.1	–	WP_110577728.1	1, 1, 1
TAIHU98	–	WP_002733640.1	–	WP_002739484.1	0, 1, 1

^a^ Letters in parentheses are domains of the referred c-di-GMP metabolism enzymes

^b^ Number of DGCs, PDEs and hybrids proteins

Bacteria encode a variety of sensory and signal transduction proteins to sense and adapt to changes in the physicochemical makeup of their environment. Sensory and signal transduction proteins encoded in the selected 24 *M. aeruginosa* genomes were predicted, and 12 sensory domain-containing proteins were found. Most of these proteins are signal transduction histidine kinases. The accession numbers and domain architectures of the highly conserved GAF, PAS, and REC domain-containing proteins are listed in Additional file 1, Table S3. As an important sensor for photosensory behavior, the GAF domain was commonly associated with c-di-GMP domains in cyanobacteria [38]. As many as 11 of the 12 proteins had the GAF domain, and some even contained two. PAS-containing proteins are related to sensory input (GAF), transduction (HAMP), or output (histidine kinases). Half of the four predicted PAS-containing proteins contain a PAC motif, a conserved region of 40–45 amino acids located at the carboxy-terminal of the PAS domain, which contributes to PAS structure [28]. Interestingly, some sensory domain-containing proteins in different genomes were identical, and were therefore assigned the same accession number, such as NIES2549 and NIES2481, DIANCHI905 and PCC7806SL, and NaRes975 and PCC9808.

### Pan-genome of *M. aeruginosa*

**Fig. 2:**
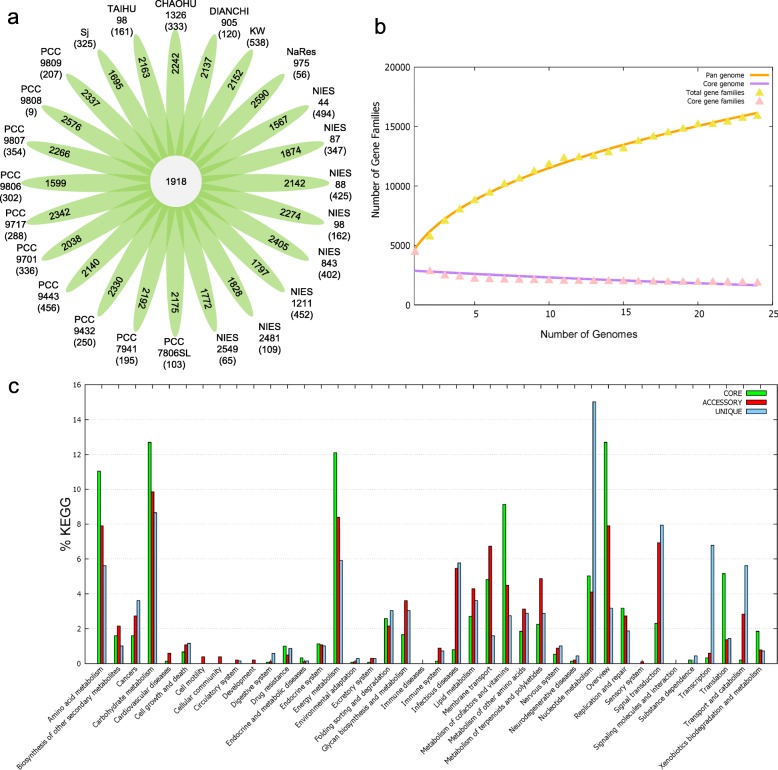
Core–pan-genome analysis of 24 *M. aeruginosa* strains by BPGA. **(a)** Flower plot diagram representing core, accessory, and unique genes of the genomes of all strains. The central circle represents the number of genes common to all strains, whereas the petals represent the number of genes in addition to the core set. Strain names are marked outside each petal and the number in brackets represents the number of unique genes corresponding to each strain. **(b)** Core–pan-genome plot for the 24 selected *M. aeruginosa* genomes. **(c)** KEGG distribution of core, accessory, and unique genes present in all 24 analyzed *M. aeruginosa* genomes

To assess the distribution of genes involved in c-di-GMP metabolism and regulation across the *M. aeruginosa* genome, a core–pan-genome analysis was performed using all 24 *M. aeruginosa* genome sequences as input in the Bacterial Pan Genome Analysis (BPGA) tool [44]. The pan-genome analysis revealed a core genome of 1918 genes with an accessory genome of 36,550 genes and 6489 unique genes (Fig. 2a). Accessory genes are those whose orthologs are present in two or more genomes, but not in all the genomes. *M. aeruginosa* possess a core genome shared by 24 strains, accounting for 37.0 to 49.9% of the genome repertoire. The core–pan plot (Fig. 2b) showed that the pan-genome trend curve did not reach a plateau and seemed to extend with addition of more genomes to the analysis. Therefore, the pan-genome was considered an “open” pan-genome. In contrast, as shown in Fig. 2b, the core genome curve leveled off, considered as a conserved core genome.

The distribution of Kyoto Encyclopedia of Genes and Genomes (KEGG) pathways and Cluster of Orthologous Groups (COG) categories in *M. aeruginosa* has been mapped in Fig. 2c and Additional file 1, Figure S1. Genes involved in c-di-GMP metabolism and regulation were assigned to gene families belonging to environmental information and signal transduction category. Among these genes, the GG[D/E]EF domain-containing DGCs and GG[D/E]EF-EAL hybrid proteins coding genes were classed into accessory genes, while the HD-GYP domain containing PDEs and the sensory genes which possess essential sensor and regulator domain, as the core genes, were relatively conservative for *M. aeruginosa* strains.

**Fig. 3:**
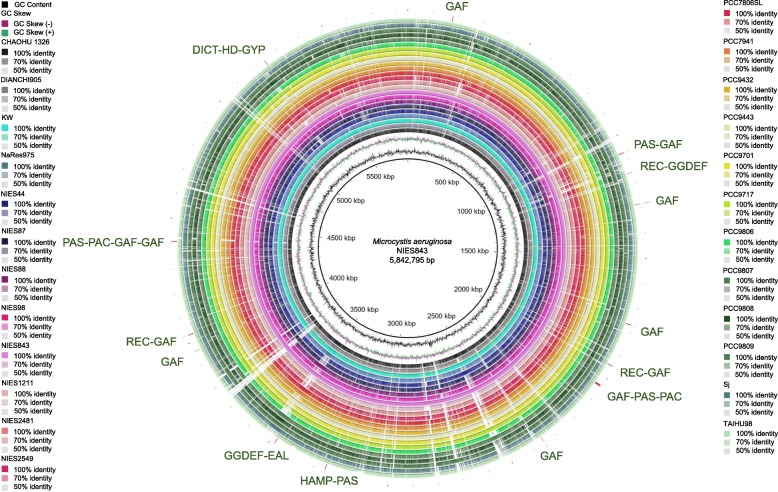
Circular visualization of 24 *M. aeruginosa* genomes by BRIG. The strain NIES843 genome was used as the reference sequence. The three inner rings show the DNA size, GC content, and GC skew of the reference genome. The 24 outer rings show regions of the comparison genomes that matched the reference genome. From inside to outside, the comparison genomes are: CHAOHU 1326, DIANCHI905, KW, NaRes975, NIES44, NIES87, NIES88, NIES98, NIES843, NIES1211, NIES2481, NIES2549, PCC7806SL, PCC7941, PCC9432, PCC9443, PCC9701, PCC9717, PCC9806, PCC9807, PCC9808, PCC9809, Sj, and TAIHU98. The location of genes encoding the domains involved in c-di-GMP signaling in the reference genome is indicated at the outer ring. The color intensity in each ring represents the BLAST match identity (%) based on the similarity to the reference genome. Gaps in the circles represent regions of low or no similarity

The BLAST Ring Image Generator (BRIG) alignment made it clear that most regions within the 24 *M. aeruginosa* genomes were conserved when compared to the reference strain NIES843 (Fig. 3). Several regions appeared to have low or even no similarity, possibly because of acquisition/deletion/rearrangement or horizontal gene transfer (HGT). The outer ring in Fig. 3 showed the distribution of genes encoding the domains involved in c-di-GMP signaling in NIES843 genome, and the specific locations were list at Additional file 1, Table S4. The corresponding sequences of other *M. aeruginosa* strains seemed highly conserved, and some even shared an identity up to 100% with the reference genome.

### Phylogenetic analysis of *M. aeruginosa* strains

**Fig. 4:**
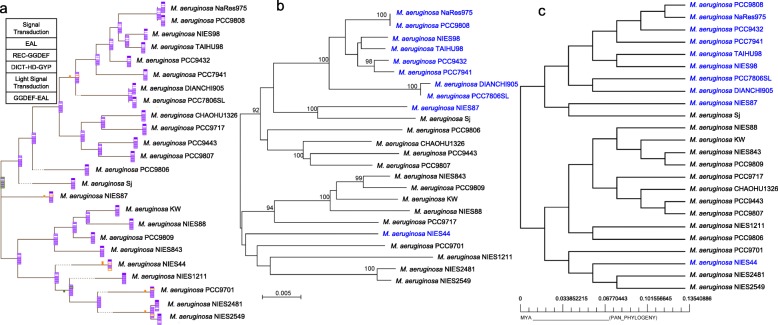
Phylogenetic analysis of *M. aeruginosa* strains. **(a)** PGL analysis of genes family involved in c-di-GMP signaling based on 31 conserved marker genes phylogenetic tree. Corresponding genes families are indicated their presence (in purple) or absence (in white) in the order as shown in the table at upper-left corner. **(b)** Maximum likelihood phylogenetic tree based on multilocus sequence analysis of concatenated conserved sensory domain sequences from 24 *M. aeruginosa* strains genomes using MEGA X. Bootstrap values above 90% are shown at the branch nodes (1000 replicates). The scale bar represents 0.005 amino acid substitutions per site. **(c)** Pan phylogenetic tree based on the pan-genome presence/absence matrix of the 24 *M. aeruginosa* genomes generated by BPGA pipeline. The scale bar at the base of the tree indicates time period in millions of years (MYA). Strains highlighted in blue represent that they do not possess REC-GGDEF domain containing DGC

Comparing with the phylogenetic tree based on the 16S rRNA gene (Additional file 1, Figure S2a), the phylogenetic tree based on the conserved marker genes, previously validated as phylogenetic markers for (cyano) bacteria [45], produced higher resolution (Fig. S2b). The 31 conserved marker genes tree revealed a topology with generally well-defined nodes, with bootstrap support values greater than 90% over 1000 replicates. Further, propensity for gene loss (PGL) analysis of the gene families revealed a group of strains have lost the REC-GGDEF domain coding gene, including strains NIES98 [46], TAIHU98, NaRes975, PCC9808, PCC9432, DIANCHI905, PCC7806SL, PCC7941, NIES87, and NIES44 (Fig. 4a). As to the node, consisting of strains NIES1211, PCC9701, NIES44, NIES2549 and NIES2481, the EAL domain coding gene seems to be acquired, but PCC9701 and NIES44 have lost this gene. Gene encoding GGDEF-EAL hybrid protein was lost in strain NIES44. There is no gain or loss of genes encoding HD-GYP and sensory domain-containing protein. To further analyze the evolution of genes encoding sensory domain-containing protein, phylogenetic tree was constructed using a multilocus sequence typing approach based on these concatenated conserved sensory domain-containing proteins sequences (Fig. 4b). It showed a similar topology with the conserved marker genes tree (Fig. S2b). Phylogenomic analyses based on binary gene presence/absence (1/0) pan-genome matrix generated by BPGA pipeline resulted in a tree (Fig. 4c) with a topology similar to the trees obtained using conserved marker genes and sensory genes (Figs. S2b, 4b). All phylogenetic trees provided more robust topologies than that based on 16S rRNA gene analysis alone.

A visual comparison of phylogenies based on 31 marker genes, sensory domains, and pan-genome presence/absence matrix were generated by tanglegram [47]. As shown in Fig. S3, only a few strains (2 of 24) occupied divergent positions on the phylogenetic trees based on 31 marker genes and sensory domains, which indicated a congruence between the two trees (Fig. S3b). Interestingly, most of that strains in which REC-GGDEF domains containing protein DGC is not detected (marked in blue), including NIES98, TAIHU98, NaRes975, PCC9808, PCC9432, DIANCHI905, PCC7806SL, and PCC7941, appeared to be phylogenetically closely related, thus were always grouped in the same clade of the different phylogenetic trees based on 31 marker genes, sensory domains, and pan-genome presence/absence matrix. Among them, DIANCHI905 and PCC7806SL [48] are representatives of toxic (microcystin-producing) bloom-forming strains; in contrast, PCC 9432 and NIES98 are non-microcystin-producing strains [49]. Specifically, pairs of strains also appeared to be phylogenetically closely related, such as NIES2549 and NIES2481, DIANCHI905 and PCC7806SL, and NaRes975 and PCC9808, although they were isolated from diverse geographic origin (Table 1). The majority of the *M. aeruginosa* strains were isolated in different locations, but no correlation was found between their geographic distribution or bloom-forming ability and phylogenetic relationships, consistent with the previous report by Meyer et al [50].

To the majority sequence of genes encoding for c-di-GMP metabolism and regulation in *M. aeruginosa*, likelihood ratio test indicated that model M2 and M8 gave a significantly better fit than model M1 and M7, respectively, which allowed individual sites to evolve under positive selection (Additional file 1, Table S5). Lots of sites with ω > 1 were identified in the sequence of genes responsible for c-di-GMP metabolism and regulation, revealing that they are likely to have been subjected to positive selection (Additional file 2, Table S6).

### Structural features of GGDEF domain, EAL domain, and HD-GYP domain of *M. aeruginosa* strains

**Fig. 5:**
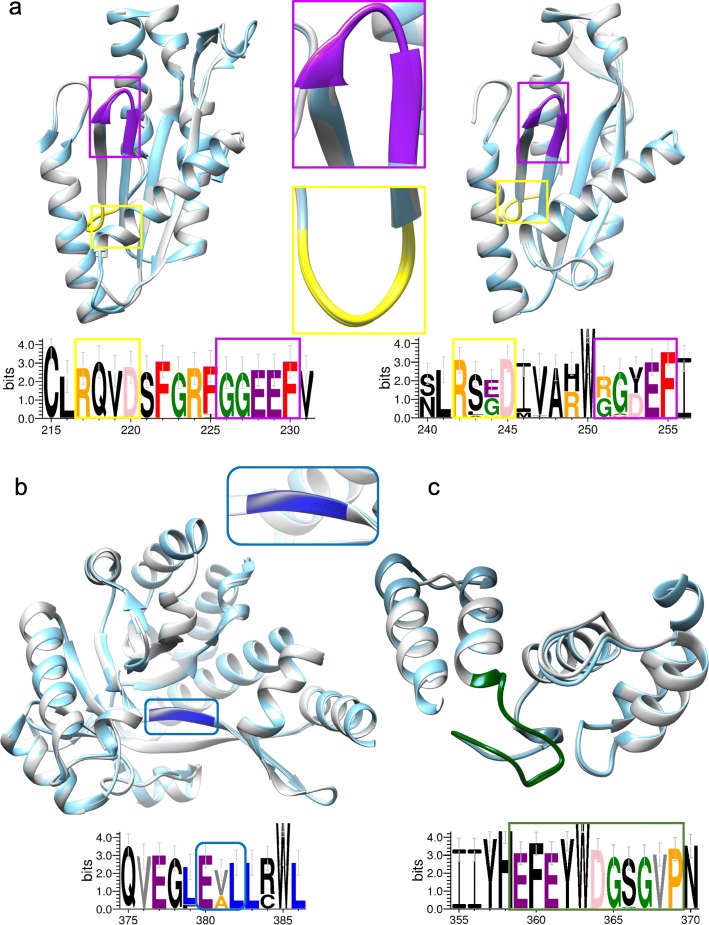
Structural features of the GG[D/E]EF and GG[D/E]EF-EAL domains from the *M. aeruginosa* NIES843 genome. The domain of NIES843 is labeled in white, and the templates are labeled in light blue. The WebLogo sequence in multiple colors represents conserved motifs from *M. aeruginosa* strains in corresponding domains. Magnifications show the structure of GG[D/E]EF, RXXD, and EAL motifs. **(a)** GG[D/E]EF domain structures from the *M. aeruginosa* NIES843 genome. Left, GGEEF domain using the crystal structure of WspR (PDB id: 3BRE) from *P. aeruginosa* as the template. Right, GG[D/E]EF domain in a hybrid protein and RmcA (PDB id: 5M3C) from *P. aeruginosa* as the template. The RXXD and GG[D/E]EF motifs are labeled in yellow and purple, respectively. **(b)** Domain structures of the EAL domain in hybrid protein from the *M. aeruginosa* NIES843 genome. RmcA (PDB id: 5M3C) from *P. aeruginosa* was used as the template. The EAL signature motif is labeled in blue. **(c)** Structures of the HD-GYP domain of the *M. aeruginosa* NIES843. PA4781 (PDB id: 4R8Z) from *P. aeruginosa* was used as the template. The GYP loop signature motif is labeled in green

To elucidate the structural features, structure predictive modeling of GGDEF domain, EAL domain, and HD-GYP domain was performed on the corresponding *M. aeruginosa* strains. The NIES843 genome is a representative genome of *M. aeruginosa* because of its genome has been completely sequenced and is modeled in Fig. 5. Similarly, the corresponding structural models of strain CHAOHU 1326 were shown in Additional file 1, Figure S4. The Z-values of the models are shown in Table S7. All the models of the GGDEF, EAL and HD-GYP domain containing proteins were qualified with a Z-score higher than − 4.0.

According to SWISS-MODEL, the crystal structure of the conserved GGDEF domain of WspR (Protein Data Bank (PDB) id: 3BRE) was selected as the template to model the structure of the GGDEF domain of the DGC [51, 52]. Amino acids S173 to N329 from the GGDEF domain were used to perform structural alignments (Fig. 5a, left). The amino acid sequences of the GGDEF domains in DGC showed a similarity of 34.2–37.2% to that of 3BRE (Additional file 1, Table S8). C-di-GMP binds to the catalytic site and to a second site distal to the catalytic loop. DGC proteins possess a conserved allosteric inhibition site (I site), composed of a RXXD motif (in which X represents any amino acid) five amino acids upstream of the GGDEF active site, that is important for controlling DGC activity. When levels of c-di-GMP are high, the second messenger can bind the RXXD motif, thereby repressing the DGC activity [53]. A systematic analysis and comparison of the 14 genomes that have corresponding GGDEF domains was performed to identify the amino acid motifs or signatures involved in catalysis and allosteric inhibition. As shown in Fig. 5a (left), the WebLogo alignment revealed that the RXXD and GGEEF motifs of the GGEEF domain were highly conserved in the same amino acid residues: Arg-Gln-Val-Asp (RQVD) and Gly-Gly-Glu-Glu-Phe (GGEEF), respectively. The GG[D/E]EF domain of the putative DGCs possessed the conserved amino acid residues essential for GTP binding, indicating that the DGCs may have catalytic activity [26].

Because only three genomes had partial EAL domains, the EAL domain in hybrid proteins from the *M. aeruginosa* NIES843 genome were chosen as paradigms to examine the crystal structure. Based on the crystal structure of the GGDEF-EAL domain of RmcA (PDB ID: 5M3C), which has a crystallographic resolution of 2.8 Å [54], the GGDEF and EAL domains in the hybrid protein of NIES843 were modeled. Compared with 5M3C, the GGDEF-EAL domains in the hybrid proteins showed sequence conservation of 35.9–37.8% (Additional file 1, Table S9). The low sequence conservation appeared to have no impact on model prediction by SWISS-MODEL. Compared with DGCs that contained only the GGDEF domain, amino acid residues of RXXD and GGDEF motifs in the GGDEF domain of the hybrid proteins were less conserved (Fig. 5a, right). The WebLogo alignment in Fig. 5b showed that amino acid residues of the EAL domain involved in the binding of c-di-GMP and catalytic activity were highly conserved in all sequences. The Glu in the EAL signature motif is an essential residue that is required to bind the c-di-GMP, whereas a change of Ala into Val (EVL) still sustains the enzymatic activity [55]. Arg in the second position downstream of the EAL signature motif was conserved in nearly all EAL domain sequences; thus, the EAL signature motif can be extended as EXLXR motif, which forms a stable platform to bind c-di-GMP [23].

Crystal structure of HD-GYP domain of *M. aeruginosa* NIES843 was modeled based on PA4781(PDB ID: 4R8Z) from *P. aeruginosa* [56] (Fig. 5c). Aligned with PA4781, the HD-GYP domain containing PDEs in *M. aeruginosa* showed sequence conservation of 33.5–34.3% (Additional file 1, Table S10). Generally, in HD-GYP domain, the HD residues clearly serve as metal ligands, the signature of HD can be extended as a larger motifs HDxGK; while the GYP motif may be more usefully considered as part of the HHExxDGxGYP, and the role of GYP motif may be substrate specificity determining but is not certainly clear [56, 57]. WebLogo alignment revealed that the GYP motifs of GYP loop in *M. aeruginosa* were highly conserved in the same amino acid residues EFExxDGxGVP, whereas Val replaced Tyr compared with the GYP motif template. Moreover, the HD motif possess YR residues in *M. aeruginosa* strains. That is, amino acid residues were replaced by YR-GVP in HD-GYP motif in all selected *M. aeruginosa* strains.

### Structural features of the PilZ domain of *M. aeruginosa* strains

All selected *M. aeruginosa* genomes encoded proteins that possess a PilZ domain. Twenty genomes encoded cellulose synthase (CelA), which contained a C-terminal PilZ domain, and the other four genomes encoded a protein that contained only a PilZ domain. The accession numbers of the corresponding proteins are shown in Additional file 1, Table S11.

**Fig. 6:**
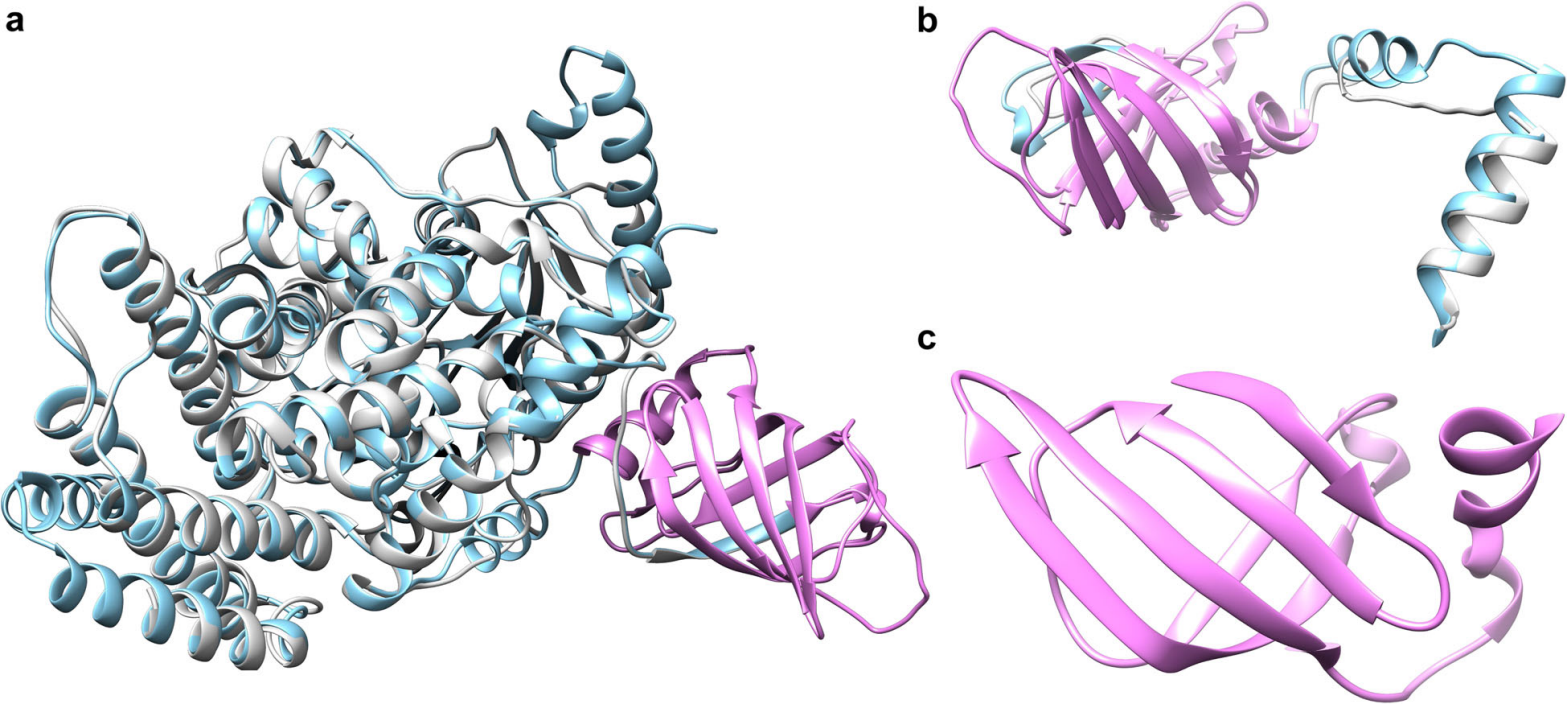
Model of the PilZ domain and PilZ-containing CelA from the *M. aeruginosa* genomes. Crystal structure of BcsA (PDB id: 4P02) from *R. sphaeroides*, the template, is labeled in light blue. The PilZ domain is labeled in pink. **(a)** PilZ-containing CelA from *M. aeruginosa* CHAOHU 1326 is labeled in white. **(b)** PilZ domain-containing protein from *M. aeruginosa* NIES843 is labeled in white. **(c)** Magnified structure of the PilZ domain

To identify the structural features, structure predictive modeling of proteins with a single PilZ domain and PilZ domain-containing CelA was performed for *M. aeruginosa* strains. Predictive modeling was based on the crystal structure of the BcsA (PDB id: 4P02) from *Rhodobacter sphaeroides*, which has a crystallographic resolution of 2.65 Å, according to SWISS-MODEL results [31]. Model of the PilZ domain-containing protein CelA of strain CHAOHU 1326 is shown in Fig. 6a. The c-di-GMP-binding PilZ domain was located in the C-terminal region of CelA and had similar structure with protein containing a single PilZ domain in Fig. 6b, which was derived from the representative *M. aeruginosa* strain NIES843. Figure 6c shows that the PilZ domain consists of a six-stranded β-barrel and a short α-helix that follows the last strand of the β-barrel.

## Discussion

The occurrence of cyanobacterial blooms appears to be increasing because of environmental factors, including continued eutrophication, rising atmospheric CO_2_ concentrations, and global warming [58–60]. Cyanobacteria can survive in ecologically diverse habitats, to a great extent, because intracellular second messengers function in pathways that mediate cellular responses to oxidative stress, nutrient imbalances, and temperature variations in the environment [8]. C-di-GMP, as a universal bacterial second messenger, has been shown to regulate biofilm formation and aggregation, which are beneficial for cyanobacteria colony formation and thus promotes bloom formation [35, 61]. With recent advances in genome sequencing and bioinformatics, it is possible to identify sequence groups with high genotypic similarity based on variation in protein-coding genes distributed across the genomes and predictions drawn from bioinformatics, and thereby provide genetic insight into c-di-GMP signaling regulation in *M. aeruginosa*. Because only one or two *M. aeruginosa* genomes do not adequately represent this species, 24 *M. aeruginosa* genomes available in NCBI’s GenBank were selected to comprehensively clarify the genetic similarities and differences of *M. aeruginosa* strains in the present study.

The selected *M. aeruginosa* strains in this study diverged to some extent at the genomic level and were isolated from aquatic ecosystems around the world. An in-depth comparative genomics analysis that included genome feature analysis, core–pan-genome analysis, and phylogenetic analysis were used to distinguish differences and similarities among the 24 selected *M. aeruginosa* genomes.

*M. aeruginosa* genome sizes result from a mix of gains and losses during natural selection as they were subjected to changing environments and competitive forces during the evolution of the species. As a freshwater species, *M. aeruginosa* have medium genomes compared with other cyanobacteria, especially compared with marine species that mostly occur in low nutrient and stable open ocean waters, such as *Synechococcus* and *Prochlorococcus*, the genome sizes of which are almost half those of *M. aeruginosa* [62]. Some reports indicated that genome size is positively correlated with the number of duplicated genes, which can originate from either within the genome itself or can be introduced by HGT [63]. Gene duplication and high genetic redundancy in the *M. aeruginosa* genomes are considered an evolutionary strategy that might confer this cyanobacterial species an extensive adaptive capacity that allows them to inhabit a wide range of habitats worldwide, and facilitates the ability to proliferate and dominate the phytoplankton communities in eutrophic freshwater ecosystems [64]. The core–pan-genome analysis indicated that these strains maintained a conserved core genome and an expansive pan-genome that allow them to acquire new genes. *M. aeruginosa* possess a relatively small core genome, which might result from high genetic diversity and variable gene content [49].

In this study, bioinformatics tools furthered our understanding of c-di-GMP signaling in *M. aeruginosa* by recognizing and studying domain architectures and tridimensional structures of the predicted proteins with DCGs, PDEs, and DGC–PDEs encoded in the genomes. These coding genes are widespread in other cyanobacterial species, such as *Synechocystis* sp. PCC6803 and *Thermosynechococcus elongatus* BP-1, which reportedly encode a considerable number of proteins predicted to be involved in c-di-GMP metabolism [36, 38]. In general, the number of domains involved in c-di-GMP signaling in cyanobacteria may be mainly determined by genome size [65]. However, there are at most three c-di-GMP signal-related domains identified in *M. aeruginosa* genomes, even if the mean genome size of this species is nearly two-fold that of the *Synechocystis* sp. PCC6803 and *Thermosynechococcus elongatus* BP-1. An alternate explanation is that, in cyanobacteria, the number of c-di-GMP signal-related domains are not simply correlated with genome size but may also be affected by bacterial adaptation. Among the species present in the CyanoBase database, the species found to lack c-di-GMP signaling systems were *Prochlorococcus* and some *Synechococcus* strains. It was reported that *Synechococcus* strains that contain c-di-GMP-modulating domains inhabit both marine and freshwater habitats and are found in rich-nutrient (eutrophic) waters, whereas *Synechococcus* strains lacking c-di-GMP-regulatory domains inhabit low-nutrient (oligotrophic) marine habitats [36]. Species adapted to stable habitats may have lost genes that encode c-di-GMP-modulating proteins. Primitive *M. aeruginosa* that inhabit low-nutrient lakes may have a small number of c-di-GMP domains even though they have relatively large genomes [66].

The domain architectures of the deduced amino acid sequences from the *M. aeruginosa* genomes also revealed diverse sensor domains, such as REC, PAS/PAC, GAF, and HAMP, which are involved in activity regulation by driving the protein dimerization process and play important roles in c-di-GMP-controlled rapid response to changing environmental conditions. It seemed that these sensor domains have been subjected to positive selection during evolution. Some sensory domain-containing proteins from different genomes have identical amino acid sequences, such as that of NIES2549 and NIES2481, DIANCHI905 and PCC7806SL, and NaRes975 and PCC9808. It should be noted that each pair of strains have close genetic relationships as determined by phylogenetic analysis.

In this study, four types of phylogenetic trees were established based on 16S rRNA gene, 31 protein-coding phylogenetic marker genes, sensory protein sequences, and binary gene presence/absence (1/0) pan matrix. The congruence of the two phylogenetic trees based on 31 protein-coding phylogenetic marker genes and sensory protein sequences facilitated a comprehensive understanding of the phylogenetic relationships and the evolution of the sensory domain coding genes among *M. aeruginosa* strains. It should be noticed that most of strains in which REC-GGDEF domains-containing protein DGC was not detected, appeared to be phylogenetically closely related. PGL analysis revealed most of genes involved in c-di-GMP signaling are stable in *M. aeruginosa* strains. In some *M. aeruginosa* strains, the EAL domain coding gene seems to be acquired from other origin by lateral gene transfer. In complex signal transduction process, the range of cellular functions might be regulated by different regulatory systems. The number of genes involved in c-di-GMP signaling might more likely rely on the number (kinds) of signals that they respond to and the intracellular level of the c-di-GMP that they need to regulate a series of physiological process, instead of genome size [67]. In some *M. aeruginosa* strains, the missing or lack of coding genes for DGC or PDE revealed that c-di-GMP-mediated regulation might not be the sole alternative regulatory pathway in this ancient photosynthetic microorganism. They might use other signal molecules such as NO, to regulate diverse biochemical and physiological processes [8].

It seems that the relatedness of the closely related strains studied did not perfectly reflect their similar physiological characteristics (e.g., cyanobacterial toxin-producing ability) or geographical origins. Phylogenetic analysis could also not reveal the *M. aeruginosa* strains with bloom-forming characteristics. Previous studies demonstrated that *Microcystis* “species” distinctions are problematic and doubtful [68, 69]. *Microcystis* taxonomic studies using 16S rRNA analysis revealed that phylogenetic trees using sequences with significantly high sequence similarities did not clearly delineate *Microcystis* species [70, 71]. In the present study, 31 protein-coding phylogenetic marker genes was used instead of discrimination based only on the traditional 16S rRNA gene, which could not sufficiently discriminate between strains [44, 45]. Similarly, the phylogenomic tree based on whole genome information was more reliable compared with the phylogenetic tree only based on the 16S rRNA gene. More tests are needed to further determine whether the alternative approaches could refine *Microcystis* species classification.

GG[D/E]EF and EAL domain-containing proteins analyzed in this study included all essential conserved amino acid residues that bind the corresponding substrate to have enzymatic activity. In *M. aeruginosa*, HD-GYP domains possess the variant key residues YR-GVP, and further study needs to be done to verify whether this domain still has the ability to bind corresponding substrates or is degenerate. Structural analysis provides important information for predicting the function of these proteins that contain GGDEF, EAL, hybrid domains, and HD-GYP domain, and creates a paradigm for future studies that analyze the evolution of enzymes involved in c-di-GMP metabolism. It was also found that all selected *M. aeruginosa* genomes encode PilZ domain, regardless of if it is in CelA, by which c-di-GMP could stimulate the biosynthesis of extracellular polysaccharides that are important for biofilm formation.

## Conclusion

In summary, comparative genomic analysis of 24 publicly available *M. aeruginosa* genomes focusing on c-di-GMP metabolism and regulation revealed the following main results:

(1) Proteins involved in c-di-GMP metabolism and regulation, such as diguanylate cyclases, phosphodiesterases, and PilZ-containing proteins, were encoded in *M. aeruginosa* genomes. However, the number of identified c-di-GMP signaling related domains was not proportional to the size of *M. aeruginosa* genomes (4.97 Mb in average). Pan-genome analysis showed that genes involved in c-di-GMP metabolism and regulation are relatively conservative in *M. aeruginosa* strains*.* (2) Phylogenetic and phylogenomic analysis revealed that the relatedness of the closely related *M. aeruginosa* strains did not reflect the geographical origins, even though they were isolated from diverse freshwater ecological environments. PGL analysis revealed that most of c-di-GMP signaling related genes are stable in *M. aeruginosa* strains. (3) In silico analysis of signaling related DGCs, PDEs, and hybrid proteins revealed that GGDEF and EAL domains contained the conserved amino acid residues essential for the substrates binding, indicating a possible catalytic activity. In addition, it was also found that all selected *M. aeruginosa* genomes encode PilZ domain, regardless of if it is in CelA.

This study is the first to analyze c-di-GMP signal-related proteins in *M. aeruginosa*, and our findings provide a pre-requisite genetic basis for further experimental characterization and evaluation of biological function. Some important aspects are still unclear that could help enhance our understanding of *M. aeruginosa* blooms in aquatic environments, such as the involvement of the specific domain-containing proteins of c-di-GMP signaling networks in *M. aeruginosa* physiological regulation and an ecologically relevant explanation of how *M. aeruginosa* adapts to its specific ecological niche.

## Methods

### *M. aeruginosa* genomes features

All of the *M. aeruginosa* genome sequences available in June 2018 in the NCBI database, annotated with the Prokaryotic Genome Annotation Pipeline [72], were used to conduct various analyses. Draft genomes that consisted of more than 1000 contigs were omitted to obtain consistent genome quality. CheckM v.1.0.8 was used to estimate the completeness of the selected *M. aeruginosa* genomes [43]. The genome features of these selected strains were listed in Table 1. The sequencing and sequence assembly of *M. aeruginosa* strain NaRes975 and CHAOHU 1326 genomes were performed as previously described [41].

### Identification of genes involved in c-di-GMP metabolism and regulation

Genes that encode the GG[D/E]EF, EAL, and GG[D/E]EF-EAL domains, the related sensor GAF, PAS, and HAMP domains, and the c-di-GMP binding domain PilZ from the selected 24 *M. aeruginosa* genome sequences were identified by performing BLAST (Identity ≥30% for amino acid and 80% for nucleotide, E-value ≤1E-5). Conserved Domain Database (CDD) [73], Microbial Signal Transduction Database (MiST, version 3.0) [74], and Simple Modular Architecture Research Tool (SMART) [75] were used to characterize the domain.

### Comparative genome analyses

Core–pan-genome analysis was performed using the BPGA tool [44]. Orthologous clusters were assigned by grouping all protein sequences encoded by the 24 genomes using the default clustering tool USEARCH based on 90% sequence identity cut-off. Core–pan-genome plots were calculated over 500 iterations. Comparative functional analysis was performed based on COG of proteins and KEGG pathways by focusing on distributions of representative protein sequences of core, accessory, and unique clusters of the *M. aeruginosa* strains. Gene families were classified accordingly. BRIG(version 0.95) [76] was used to create a circular genome comparison to highlight the location of genes encoding c-di-GMP-associated signaling domains between the 24 genomes compared with the reference sequence.

### Phylogeny and evolution analysis

To elucidate the phylogenetic relationships between the *M. aeruginosa* strains, 16S rRNA gene sequences of cyanobacterial strains for which whole genome sequence data were available on NCBI were downloaded and analyzed to construct a phylogenetic tree. Sequences were aligned in MUSCLE version 3.8 with default settings [77]; then, the phylogenetic and molecular evolutionary analyses were conducted using MEGA version X [78]. The phylogenetic tree was inferred using the neighbor-joining method with 1000 bootstrap replications. The evolutionary distances were computed using the maximum composite likelihood method and the units were number of base substitutions per site. The analysis involved 25 nucleotide sequences, including 24 sequences of *M. aeruginosa* strains and the *Synechocystis* sp. PCC6803 sequence as the outgroup. All positions that contained gaps and missing data were eliminated. There were a total of 1313 nucleotides in the final dataset.

To assess relationships between the *M. aeruginosa* strains, the phylogenetic tree was constructed based on amino acid sequences of 31 highly conserved proteins that were encoded by the genes distributed in genomes as a single copy along 24 *M. aeruginosa* genomes, according Wu and Eisen (2008) [45, 79]. These protein sequences were mined by the AutoMated Phylogenomic inference Application−AMPHORA2 tool [80, 81], using default settings for the bacteria option and a cut-off E-value of 1 E-10. Individual alignments were performed for each of the 31 gene sets in MUSCLE version 3.8 with default settings [77], trimmed with respect to the reading frame, and subsequently concatenated with the FaBox Fasta Alignment Joiner [82]. Only genomes with all selected sets of conserved genes were used in the phylogenetic analysis. A Maximum Likelihood (ML) tree was constructed with MEGA X using the Jones–Taylor–Thornton model with nearest neighbor interchange [78, 83]. Then, 1000 bootstrap replicates were calculated to evaluate relative branch support. There were 7481 total nucleotides in the final dataset. After that, the program Count was performed to compute PGL to quantify the frequency of loss for select gene family of the key node in the 31 marker genes phylogenetic tree [84].

A multilocus sequence typing approach based on concatenation of sensory domain containing proteins, was used to generate the phylogenetic reconstructed tree following protocols described. Individual alignments were performed for each of the sensory genes in MUSCLE version 3.8 with default settings, trimmed with respect to the reading frame, and subsequently concatenated with the FaBox Fasta Alignment Joiner [82]. ML tree was constructed with MEGA X using the Jones–Taylor–Thornton model and a bootstrap resampling value of 1000. There were 4951 total nucleotides in the final dataset.

The pan phylogenetic tree was reconstructed using the neighbor-joining algorithm based on a binary gene presence/absence (1/0) pan matrix generated by BPGA from orthologous clusters after clustered by USEARCH. The trees in Newick format were then loaded into Dendroscope 3 and the tanglegram algorithm was applied for further comparison [47, 85].

Nucleotide sequences of c-di-GMP signal-related genes were aligned by MUSCLE version 3.8 with default settings [77]. Phylogenetic trees files were generated by MEGA version X with ML model [78]. Aligned sequences in conjunction with ML tree files were used as input for evolution analysis using codeml from the PAML (version 4.9) with site models [86]. Likelihood ratio test was performed among pairs of models (M1 and M2; M7 and M8).

### Proteins structural analyses

Automated protein structure models were predicted and built by the SWISS-MODEL server [51] by searching for evolutionarily related protein structures against the SWISS-MODEL template library SMTL based on the PDB database [87, 88]. In this platform, templates are ranked based on the expected quality of the resulting models, and estimated by Global Model Quality Estimate and Quaternary Structure Quality Estimate [88, 89]. The crystal structures of a DGC (WspR) from *P. aeruginosa* [52], the GG[D/E]EF-EAL hybrid domain protein RmcA from *P. aeruginosa* [54], the HD-GYP domain containing protein PA4781 from *P. aeruginosa* [56], and the PilZ domain-containing protein BcsA from *R. sphaeroides* were selected as templates for the structural analyses [31]. QMEAN scoring functions were used to estimate alternative models and screen for models whose scores strongly matched high-resolution structures that were then used to create the corresponding model [90]. Structures were matched using Chimera UCSF [91]. CDD was used to identify the amino acids of the motifs present in the various domains and PROSITE was used to determine the site [92]. Multiple protein sequence alignments were generated through MUSCLE with default parameters. Conserved motif sequence figures were visualized using WebLogo based on aligned amino acid sequences [93].

## Supplementary information

**Additional file 1: Figure S1.** COG distribution of core, accessory and unique genes present in 24 analyzed *M. aeruginosa* genomes. **Figure S2.** Phylogenetic analysis of *M. aeruginosa* strains. **(a)** Neighbour-joining phylogenetic tree based on the 16S rRNA gene sequences of 25 genomes. Twenty-five strains are used in this study plus *Synechocystis* sp. PCC 6803 as the outgroup. Bootstrap values above 90% are shown at the branch nodes (1000 replicates). The scale bar represents 0.01 nucleotide substitutions per site. **(b)** Maximum likelihood phylogenetic tree based on multilocus sequence analysis of 31 concatenated conserved marker genes from 24 *M. aeruginosa* strains genomes using MEGA X. Bootstrap values above 90% are shown at the branch nodes (1000 replicates). The scale bar represents 0.02 amino acid substitutions per site. Strains highlighted in blue represent that they do not possess REC-GGDEF domain containing DGC. **Figure S3.** Tanglegram comparison of the phylogenetic trees. **(a)** The 31 marker genes tree (left) is compared with pan-genome phylogenomic tree (right). **(b)** The 31 marker genes tree (left) is compared with one generated using sensor genes tree (right). **(c)** The sensor genes tree (left) is compared with pan-genome phylogenomic tree (right). **Figure S4.** Structural features of GGDEF domain, and GGDEF-EAL domain from the *M. aeruginosa* CHAOHU1326 genome. The domain surface of CHAOHU 1326 are labeled in white, the templates are labeled in light blue. **(a)** GGDEF domain structures from the *M. aeruginosa* CHAOHU 1326 genome. Left, GGDEF domain taking crystal structure of WspR (PDB id: 3BRE) from *P. aeruginosa* as template. Right, GGDEF domain in hybrid protein, and RmcA (PDB id: 5M3C) from *P. aeruginosa* is used as template. **(b)** Domain structures of EAL domain in hybrid protein from the *M. aeruginosa* CHAOHU 1326 genome. RmcA (PDB id: 5M3C) from *P. aeruginosa* is used as template. The RXXD, GGEEF and EAL signature motif are labeled in yellow, purple and blue, respectively. **(c)** Structures of the HD-GYP domain of the *M. aeruginosa* CHAOHU1326. PA4781 (PDB id: 4R8Z) from *P. aeruginosa* was used as the template. The GYP loop signature motif is labeled in green. **Table S1.** Genome features of *M. aeruginosa* CHAOHU 1326 and NaRes975. **Table S2.** Numbers of RNA genes found in all 24 analyzed *M. aeruginosa* genomes. **Table S3.** Highly conserved GAF and PAS domain-containing protein accession numbers and domain architectures in *M. aeruginosa*. **Table S4.** Locations of genes related to c-di-GMP metabolism and regulation in *M. aeruginosa* NIES843. **Table S5.** Positive selection for genes related to c-di-GMP metabolism and regulation in *M. aeruginosa*. **Table S7.** QMEAN Z-score of the predicted structures of EAL, GGDEF, HD-GYP and PilZ domain containing proteins. **Table S8.** Identity of DGC sequences from *M. aeruginosa* genomes compared to WspR from *P. aeruginosa*. **Table S9**. Identity of GGDEF-EAL domain sequences from *M. aeruginosa* genomes compared to that from *P. aeruginosa*. **Table S10**. Identity of HD-GYP containing PDE sequences from *M. aeruginosa* genomes compared to that from *P. aeruginosa*. **Table S11.** Accession numbers of the predicted PilZ containing proteins found in 24 analyzed *M. aeruginosa* genomes.

**Additional file 2: Table S6.** Positive selection of genes related to c-di-GMP metabolism and regulation in *M. aeruginosa.*

## Data Availability

All genome sequences analyzed in the current study are available on the NCBI GenBank database under the accession numbers as provided in Table 1. Specifically, the whole genome sequence of *M. aeruginosa* CHAOHU 1326 and NaRes975 have been deposited in the GenBank database under accession number MOLZ00000000 and MOLN00000000, respectively. All data generated during this study are included within the paper and/or additional files.

## Abbreviations

BPGA: Bacterial Pan Genome Analysis
BRIG: BLAST Ring Image Generator
CDD: Conserved Domain database
c-di-GMP: Bis-(3′,5′)-cyclic-dimeric-guanosine monophosphate
CDSs: Protein-coding sequences
COG: Cluster of Orthologous Groups
DGCs: Diguanylate cyclases
FHA: Forkhead associated
GAF: cGMP phosphodiesterase/adenylyl cyclase/FhlA
HAMP: Histidine kinases/adenylate cyclases/methyl accepting proteins and phosphatases
HGT: Horizontal gene transfer
KEGG: Kyoto Encyclopedia of Genes and Genomes
MiST: Microbial Signal Transduction Database
ML: Maximum Likelihood
NCBI: National Center for Biotechnology Information
ncRNA: Noncoding RNA
PAS: Per/Arnt/Sim
PDB: Protein Data Bank
PDEs: Phosphodiesterases
PGL: Propensity for gene loss
pGpG: 5′-phosphoguanylyl-(3′-5′)-guanosine
REC: Response regulator receiver
SMART: Simple Modular Architecture Research Tool

## References

[1] Harke MJ, Steffen MM, Gobler CJ, Otten TG, Wilhelm SW, Wood SA (2016). A review of the global ecology, genomics, and biogeography of the toxic cyanobacterium, *Microcystis* spp. Harmful Algae.

[2] Garcia-Pichel F, Belnap J, Neuer S, Schanz F (2003). Estimates of global cyanobacterial biomass and its distribution. Arch Hydrobiol Suppl Algol Stud.

[3] Paerl HW, Huisman J (2008). Blooms like it hot. Science.

[4] Huisman J, Codd GA, Paerl HW, Ibelings BW, Verspagen JMH, Visser PM (2018). Cyanobacterial blooms. Nat Rev Microbiol.

[5] MacKintosh C, Beattie KA, Klumpp S, Cohen P, Codd GA (1990). Cyanobacterial microcystin-LR is a potent and specific inhibitor of protein phosphatases 1 and 2A from both mammals and higher plants. FEBS Lett.

[6] Yoshizawa S, Matsushima R, Watanabe MF, Harada K, Ichihara A, Carmichael WW (1990). Inhibition of protein phosphatases by microcystins and nodularin associated with hepatotoxicity. J Cancer Res Clin Oncol.

[7] Codd GA, Lindsay J, Young FM, Morrison LF, Metcalf JS, Huisman J, Matthijs HCP, Visser PM (2005). Harmful Cyanobacteria. Harmful cyanobacteria.

[8] Agostoni M, Montgomery BL (2014). Survival strategies in the aquatic and terrestrial world: the impact of second messengers on cyanobacterial processes. Life Basel.

[9] Townsley L, Yildiz FH (2015). Temperature affects c-di-GMP signalling and biofilm formation in *Vibrio cholerae*. Environ Microbiol.

[10] Valentini M, Filloux A (2016). Biofilms and cyclic di-GMP (c-di-GMP) signaling: lessons from *Pseudomonas aeruginosa* and other bacteria. J Biol Chem.

[11] An SW, Wu JE, Zhang LH (2010). Modulation of *Pseudomonas aeruginosa* biofilm dispersal by a cyclic-di-GMP phosphodiesterase with a putative hypoxia-sensing domain. Appl Environ Microbiol.

[12] Römling U, Galperin MY, Gomelsky M (2013). Cyclic di-GMP: the first 25 years of a universal bacterial second messenger. Microbiol Mol Biol Rev.

[13] Sauer K, Walker JM (2017). c-di-GMP Signaling.

[14] Boyd Chelsea D., O'Toole George A. (2012). Second Messenger Regulation of Biofilm Formation: Breakthroughs in Understanding c-di-GMP Effector Systems. Annual Review of Cell and Developmental Biology.

[15] Duerig A, Abel S, Folcher M, Nicollier M, Schwede T, Amiot N (2009). Second messenger-mediated spatiotemporal control of protein degradation regulates bacterial cell cycle progression. Genes Dev.

[16] He Y-W, Zhang L-H (2008). Quorum sensing and virulence regulation in *Xanthomonas campestris*. FEMS Microbiol Rev.

[17] Liang ZX (2015). The expanding roles of c-di-GMP in the biosynthesis of exopolysaccharides and secondary metabolites. Nat Prod Rep.

[18] Ryjenkov DA, Tarutina M, Moskvin OV, Gomelsky M (2005). Cyclic diguanylate is a ubiquitous signaling molecule in bacteria: insights into biochemistry of the GGDEF protein domain. J Bacteriol.

[19] Chan C, Paul R, Samoray D, Amiot NC, Giese B, Jenal U (2004). Structural basis of activity and allosteric control of diguanylate cyclase. Proc Natl Acad Sci U S A.

[20] Whiteley CG, Lee DJ (2015). Bacterial diguanylate cyclases: structure, function and mechanism in exopolysaccharide biofilm development. Biotechnol Adv.

[21] Sultan SZ, Pitzer JE, Boquoi T, Hobbs G, Miller MR, Motaleb MA (2011). Analysis of the HD-GYP domain cyclic dimeric GMP phosphodiesterase reveals a role in motility and the enzootic life cycle of *Borrelia burgdorferi*. Infect Immun.

[22] Christen M, Christen B, Folcher M, Schauerte A, Jenal U (2005). Identification and characterization of a cyclic di-GMP-specific phosphodiesterase and its allosteric control by GTP. J Biol Chem.

[23] Chou SH, Galperin MY (2016). Diversity of cyclic di-GMP-binding proteins and mechanisms. J Bacteriol.

[24] Navarro M, De N, Bae N, Wang Q, Sondermann H (2009). Structural analysis of the GGDEF-EAL domain-containing c-di-GMP receptor FimX. Structure.

[25] Galperin MY (2010). Diversity of structure and function of response regulator output domains. Curr Opin Microbiol.

[26] Mata AR, Pacheco CM, Cruz Pérez JF, Sáenz MM, Baca BE (2018). In silico comparative analysis of GGDEF and EAL domain signaling proteins from the *Azospirillum* genomes. BMC Microbiol.

[27] Römling U, Galperin MY, Gomelsky M (2013). Distribution of GGDEF, EAL, HD-GYP and PilZ domains in bacterial genomes.

[28] Henry JT, Crosson S (2011). Ligand-binding PAS domains in a genomic, cellular, and structural context. Annu Rev Microbiol.

[29] Schirmer T (2016). C-di-GMP synthesis: structural aspects of evolution, catalysis and regulation. J Mol Biol.

[30] Szklarczyk D, Gable AL, Lyon D, Junge A, Wyder S, Huerta-Cepas J (2019). STRING v11: protein-protein association networks with increased coverage, supporting functional discovery in genome-wide experimental datasets. Nucleic Acids Res.

[31] Morgan JLW, McNamara JT, Zimmer J (2014). Mechanism of activation of bacterial cellulose synthase by cyclic di-GMP. Nat Struct Mol Biol.

[32] Amikam D, Galperin MY (2006). PilZ domain is part of the bacterial c-di-GMP binding protein. Bioinformatics.

[33] Schäper S, Steinchen W, Krol E, Altegoer F, Skotnicka D, Søgaard-Andersen L (2017). AraC-like transcriptional activator CuxR binds c-di-GMP by a PilZ-like mechanism to regulate extracellular polysaccharide production. Proc Natl Acad Sci U S A.

[34] Fujiwara T, Komoda K, Sakurai N, Tajima K, Tanaka I, Yao M (2013). The c-di-GMP recognition mechanism of the PilZ domain of bacterial cellulose synthase subunit a. Biochem Biophys Res Commun.

[35] Agostoni M, Waters CM, Montgomery BL (2016). Regulation of biofilm formation and cellular buoyancy through modulating intracellular cyclic di-GMP levels in engineered cyanobacteria. Biotechnol Bioeng.

[36] Agostoni M, Koestler BJ, Waters CM, Williams BL, Montgomery BL (2013). Occurrence of cyclic di-GMP-modulating output domains in cyanobacteria: an illuminating perspective. mBio.

[37] Gen E, Ryouhei N, Takashi S, Rei N, Masahiko I (2014). Cyanobacteriochrome SesA is a diguanylate cyclase that induces cell aggregation in *Thermosynechococcus*. J Biol Chem.

[38] Savakis P, De CS, Angerer V, Ruppert U, Anders K, Essen LO (2012). Light-induced alteration of c-di-GMP level controls motility of *Synechocystis* sp. PCC 6803. Mol Microbiol.

[39] Yamaguchi H, Suzuki S, Osana Y, Kawachi M (2018). Complete genome sequence of *Microcystis aeruginosa* NIES-2481 and common genomic features of group G *M. aeruginosa*. J Genomics.

[40] Yamaguchi H, Suzuki S, Tanabe Y, Osana Y, Shimura Y, Ishida K-I, et al. Complete genome sequence of *Microcystis aeruginosa* NIES-2549, a bloom-forming cyanobacterium from Lake Kasumigaura, Japan. Genome Announc. 2015;3(3):e00551–15.10.1128/genomeA.00551-15. 10.1128/genomeA.00551-15 PMC4447913 26021928

[41] Chen M, Tian L, Ren C, Xu C, Wang Y, Li L (2019). Extracellular polysaccharide synthesis in a bloom-forming strain of *Microcystis aeruginosa*: implications for colonization and buoyancy. Sci Rep.

[42] Tian L, Chen M, Ren C, Wang Y, Li L (2018). Anticyanobacterial effect of l-lysine on *Microcystis aeruginosa*. RSC Adv.

[43] Parks DH, Imelfort M, Skennerton CT, Hugenholtz P, Tyson GW (2015). CheckM: assessing the quality of microbial genomes recovered from isolates, single cells, and metagenomes. Genome Res.

[44] Chaudhari NM, Gupta VK, Dutta C (2016). BPGA- an ultra-fast pan-genome analysis pipeline. Sci Rep.

[45] Wu M, Eisen JA (2008). A simple, fast, and accurate method of phylogenomic inference. Genome Biol.

[46] Yamaguchi H, Suzuki S, Sano T, Tanabe Y, Nakajima N, Kawachi M (2016). Draft genome sequence of *Microcystis aeruginosa* NIES-98, a non-microcystin-producing cyanobacterium from Lake Kasumigaura, Japan. Genome Announc.

[47] Scornavacca C, Zickmann F, Huson DH (2011). Tanglegrams for rooted phylogenetic trees and networks. Bioinformatics.

[48] Frangeul L, Quillardet P, Castets A-M, Humbert J-F, Matthijs HCP, Cortez D (2008). Highly plastic genome of *Microcystis aeruginosa* PCC 7806, a ubiquitous toxic freshwater cyanobacterium. BMC Genomics.

[49] Pérez-Carrascal OM, Terrat Y, Giani A, Fortin N, Greer CW, Tromas N (2019). Coherence of *Microcystis* species revealed through population genomics. ISME J.

[50] Meyer KA, Davis TW, Watson SB, Denef VJ, Berry MA, Dick GJ (2017). Genome sequences of lower Great Lakes *Microcystis* sp. reveal strain-specific genes that are present and expressed in western Lake Erie blooms. PLoS One.

[51] Waterhouse A, Bertoni M, Bienert S, Studer G, Tauriello G, Gumienny R (2018). SWISS-MODEL: homology modelling of protein structures and complexes. Nucleic Acids Res.

[52] De N, Pirruccello M, Krasteva PV, Bae N, Raghavan RV, Sondermann H (2008). Phosphorylation-independent regulation of the diguanylate cyclase WspR. PLoS Biol.

[53] Chen MW, Kotaka M, Vonrhein C, Bricogne G, Rao F, Chuah MLC (2012). Structural insights into the regulatory mechanism of the response regulator RocR from *Pseudomonas aeruginosa* in cyclic Di-GMP signaling. J Bacteriol.

[54] Mantoni F, Paiardini A, Brunotti P, D'Angelo C, Cervoni L, Paone A (2018). Insights into the GTP-dependent allosteric control of c-di-GMP hydrolysis from the crystal structure of PA0575 protein from *Pseudomonas aeruginosa*. FEBS J.

[55] Rao F, Yang Y, Qi Y, Liang Z-X (2008). Catalytic mechanism of cyclic di-GMP-specific phosphodiesterase: a study of the EAL domain-containing RocR from *Pseudomonas aeruginosa*. J Bacteriol.

[56] Rinaldo Serena, Paiardini Alessandro, Stelitano Valentina, Brunotti Paolo, Cervoni Laura, Fernicola Silvia, Protano Carmela, Vitali Matteo, Cutruzzolà Francesca, Giardina Giorgio (2015). Structural Basis of Functional Diversification of the HD-GYP Domain Revealed by the Pseudomonas aeruginosa PA4781 Protein, Which Displays an Unselective Bimetallic Binding Site. Journal of Bacteriology.

[57] Ryan RP, Dow JM (2010). Intermolecular interactions between HD-GYP and GGDEF domain proteins mediate virulence-related signal transduction in *Xanthomonas campestris*. Virulence.

[58] Ullah H, Nagelkerken I, Goldenberg SU, Fordham DA (2018). Climate change could drive marine food web collapse through altered trophic flows and cyanobacterial proliferation. PLoS Biol.

[59] Visser PM, Verspagen JMH, Sandrini G, Stal LJ, Matthijs HCP, Davis TW (2016). How rising CO2 and global warming may stimulate harmful cyanobacterial blooms. Harmful Algae.

[60] O’Neil JM, Davis TW, Burford MA, Gobler CJ (2012). The rise of harmful cyanobacteria blooms: the potential roles of eutrophication and climate change. Harmful Algae.

[61] Rossi F, De Philippis R (2015). Role of cyanobacterial exopolysaccharides in phototrophic biofilms and in complex microbial mats. Life Basel.

[62] Bentkowski P, Oosterhout CV, Ashby B, Mock T (2017). The effect of extrinsic mortality on genome size evolution in prokaryotes. ISME J.

[63] Humbert JF, Barbe V, Latifi A, Gugger M, Calteau A, Coursin T (2013). A tribute to disorder in the genome of the bloom-forming freshwater Cyanobacterium *Microcystis aeruginosa*. PLoS One.

[64] Larsson J, Nylander JAA, Bergman B (2011). Genome fluctuations in cyanobacteria reflect evolutionary, developmental and adaptive traits. BMC Evol Biol.

[65] Römling U, Liang Z-X, Dow JM (2017). Progress in understanding the molecular basis underlying functional diversification of cyclic dinucleotide turnover proteins. J Bacteriol.

[66] Wilson AE, Sarnelle O, Neilan BA, Salmon TP, Gehringer MM, Hay ME (2005). Genetic variation of the bloom-forming Cyanobacterium *Microcystis aeruginosa* within and among lakes: implications for harmful algal blooms. Appl Environ Microbiol.

[67] Seshasayee ASN, Fraser GM, Luscombe NM (2010). Comparative genomics of cyclic-di-GMP signalling in bacteria: post-translational regulation and catalytic activity. Nucleic Acids Res.

[68] Šejnohová L, Maršálek B, Whitton BA (2012). Microcystis. Ecology of cyanobacteria II: their diversity in space and time.

[69] Lyra C, Suomalainen S, Gugger M, Vezie C, Sundman P, Paulin L (2001). Molecular characterization of planktic cyanobacteria of *Anabaena*, *Aphanizomenon*, *Microcystis* and *Planktothrix* genera. Int J Syst Evol Microbiol.

[70] Otsuka S, Suda S, Li R, Watanabe M, Oyaizu H, Matsumoto S (1998). 16S rDNA sequences and phylogenetic analyses of *Microcystis* strains with and without phycoerythrin. J Fems Microbiol Lett.

[71] Otsuka S, Suda S, Shibata S, Oyaizu H, Matsumoto S, Watanabe M (2001). A proposal for the unification of five species of the cyanobacterial genus *Microcystis* Kützing *ex* Lemmermann 1907 under the rules of the bacteriological code. Int J Syst Evol Microbiol.

[72] Badretdin A, Nawrocki EP, Ostell J, Pruitt KD, Zaslavsky L, DiCuccio M (2016). NCBI prokaryotic genome annotation pipeline. Nucleic Acids Res.

[73] Marchlerbauer A, Bo Y, Han L, He J, Lanczycki CJ, Lu S (2017). CDD/SPARCLE: functional classification of proteins via subfamily domain architectures. Nucleic Acids Res.

[74] Ulrich LE, Zhulin IB (2010). The MiST2 database: a comprehensive genomics resource on microbial signal transduction. Nucleic Acids Res.

[75] Letunic I, Bork P (2018). 20 years of the SMART protein domain annotation resource. Nucleic Acids Res.

[76] Alikhan N-F, Petty NK, Ben Zakour NL, Beatson SA (2011). BLAST ring image generator (BRIG): simple prokaryote genome comparisons. BMC Genomics.

[77] Edgar RC (2004). MUSCLE: multiple sequence alignment with high accuracy and high throughput. Nucleic Acids Res.

[78] Kumar Sudhir, Stecher Glen, Li Michael, Knyaz Christina, Tamura Koichiro (2018). MEGA X: Molecular Evolutionary Genetics Analysis across Computing Platforms. Molecular Biology and Evolution.

[79] Shih PM, Dongying W, Amel L, Axen SD, Fewer DP, Emmanuel T (2013). Improving the coverage of the cyanobacterial phylum using diversity-driven genome sequencing. Proc Natl Acad Sci U S A.

[80] Wu M, Scott AJ (2012). Phylogenomic analysis of bacterial and archaeal sequences with AMPHORA2. Bioinformatics.

[81] Kerepesi C, Bánky D, Grolmusz V (2014). AmphoraNet: the webserver implementation of the AMPHORA2 metagenomic workflow suite. Gene.

[82] Villesen P (2007). FaBox: an online toolbox for fasta sequences. Mol Ecol Notes.

[83] Jones DT, Taylor WR, Thornton JM (1992). The rapid generation of mutation data matrices from protein sequences. Comput Appl Biosci.

[84] Csuos M. (2010). Count: evolutionary analysis of phylogenetic profiles with parsimony and likelihood. Bioinformatics.

[85] Huson DH, Scornavacca C (2012). Dendroscope 3: an interactive tool for rooted phylogenetic trees and networks. Syst Biol.

[86] Yang Z. (2007). PAML 4: Phylogenetic Analysis by Maximum Likelihood. Molecular Biology and Evolution.

[87] Berman HM, Battistuz T, Bhat TN, Bluhm WF, Bourne PE, Burkhardt K (2002). The protein data Bank. Acta Crystallogr Sect D Struct Biol.

[88] Biasini M, Bienert S, Waterhouse A, Arnold K, Studer G, Schmidt T (2014). SWISS-MODEL: modelling protein tertiary and quaternary structure using evolutionary information. Nucleic Acids Res.

[89] Bertoni M, Kiefer F, Biasini M, Bordoli L, Schwede T. Modeling protein quaternary structure of homo- and hetero-oligomers beyond binary interactions by homology. Sci Rep. 2017;7(1):–10480. 10.1038/s41598-017-09654-8. 10.1038/s41598-017-09654-8 PMC5585393 28874689

[90] Benkert P, Biasini M, Schwede T (2011). Toward the estimation of the absolute quality of individual protein structure models. Bioinformatics (Oxford, England).

[91] Pettersen Eric F., Goddard Thomas D., Huang Conrad C., Couch Gregory S., Greenblatt Daniel M., Meng Elaine C., Ferrin Thomas E. (2004). UCSF Chimera?A visualization system for exploratory research and analysis. Journal of Computational Chemistry.

[92] Sigrist CJA, de Castro E, Cerutti L, Cuche BA, Hulo N, Bridge A (2013). New and continuing developments at PROSITE. Nucleic Acids Res.

[93] Crooks GE, Hon G, Chandonia JM, Brenner SE (2004). WebLogo: A sequence logo generator. Genome Res.

