# Supplementary material for: Comparative genomics analysis of c-di-GMP metabolism and regulation in Microcystis aeruginosa
Source: BMC Genomics. 2020 Mar 9;21:217. doi: 10.1186/s12864-020-6591-3 (PMC7063779; doi:10.1186/s12864-020-6591-3)
Supplement: Supplementary file 1 — Additional file 1: Figure S1. COG distribution of core, accessory and unique genes present in 24 analyzed M. aeruginosa genomes. Figure S2. Phylogenetic analysis of M. aeruginosa strains. (a) Neighbour-joining phylogenetic tree based on the 16S rRNA gene sequences of 25 genomes. Twenty-five strains are used in this study plus Synechocystis sp. PCC 6803 as the outgroup. Bootstrap values above 90% are shown at the branch nodes (1000 replicates). The scale bar represents 0.01 nucleotide substitutions per site. (b) Maximum likelihood phylogenetic tree based on multilocus sequence analysis of 31 concatenated conserved marker genes from 24 M. aeruginosa strains genomes using MEGA X. Bootstrap values above 90% are shown at the branch nodes (1000 replicates). The scale bar represents 0.02 amino acid substitutions per site. Strains highlighted in blue represent that they do not possess REC-GGDEF domain containing DGC. Figure S3. Tanglegram comparison of the phylogenetic trees. (a) The 31 marker genes tree (left) is compared with pan-genome phylogenomic tree (right). (b) The 31 marker genes tree (left) is compared with one generated using sensor genes tree (right). (c) The sensor genes tree (left) is compared with pan-genome phylogenomic tree (right). Figure S4. Structural features of GGDEF domain, and GGDEF-EAL domain from the M. aeruginosa CHAOHU1326 genome. The domain surface of CHAOHU 1326 are labeled in white, the templates are labeled in light blue. (a) GGDEF domain structures from the M. aeruginosa CHAOHU 1326 genome. Left, GGDEF domain taking crystal structure of WspR (PDB id: 3BRE) from P. aeruginosa as template. Right, GGDEF domain in hybrid protein, and RmcA (PDB id: 5M3C) from P. aeruginosa is used as template. (b) Domain structures of EAL domain in hybrid protein from the M. aeruginosa CHAOHU 1326 genome. RmcA (PDB id: 5M3C) from P. aeruginosa is used as template. The RXXD, GGEEF and EAL signature motif are labeled in yellow, purple and blue, respectively. (c) St [file 12864_2020_6591_MOESM1_ESM.docx]

**Comparative genomics analysis** **of c-di-GMP metabolism and regulation in** ***Microcystis aeruginosa***

**Authors**

Meng Chen^a^, Chun-Yang Xu^a^, Xu Wang^a^, Chong-Yang Ren^a^, Jiao Ding^a^, Li Li*^a,b^

**Affiliation**

^a^ Shandong Provincial Key Laboratory of Water Pollution Control and Resource Reuse, School of Environmental Science and Engineering, Shandong University, Qingdao, China

^b^ Shandong Provincial Engineering Center on Environmental Science and Technology, Jinan, China

***Corresponding author**

**E-mails**

Meng Chen: [chenmeng921220@163.com](mailto:chenmeng921220@163.com);

Chun-Yang Xu: [chunyang9612@163.com](mailto:chunyang9612@163.com);

Xu Wang: [wangxuhappyya@163.com](mailto:wangxuhappyya@163.com);

Chong-Yang Ren: [rency0509@163.com](mailto:rency0509@163.com);

Jiao Ding: [dingjiao0317@foxmail.com](mailto:dingjiao0317@foxmail.com);

Li Li: [lili@sdu.edu.cn](mailto:lili@sdu.edu.cn)

**
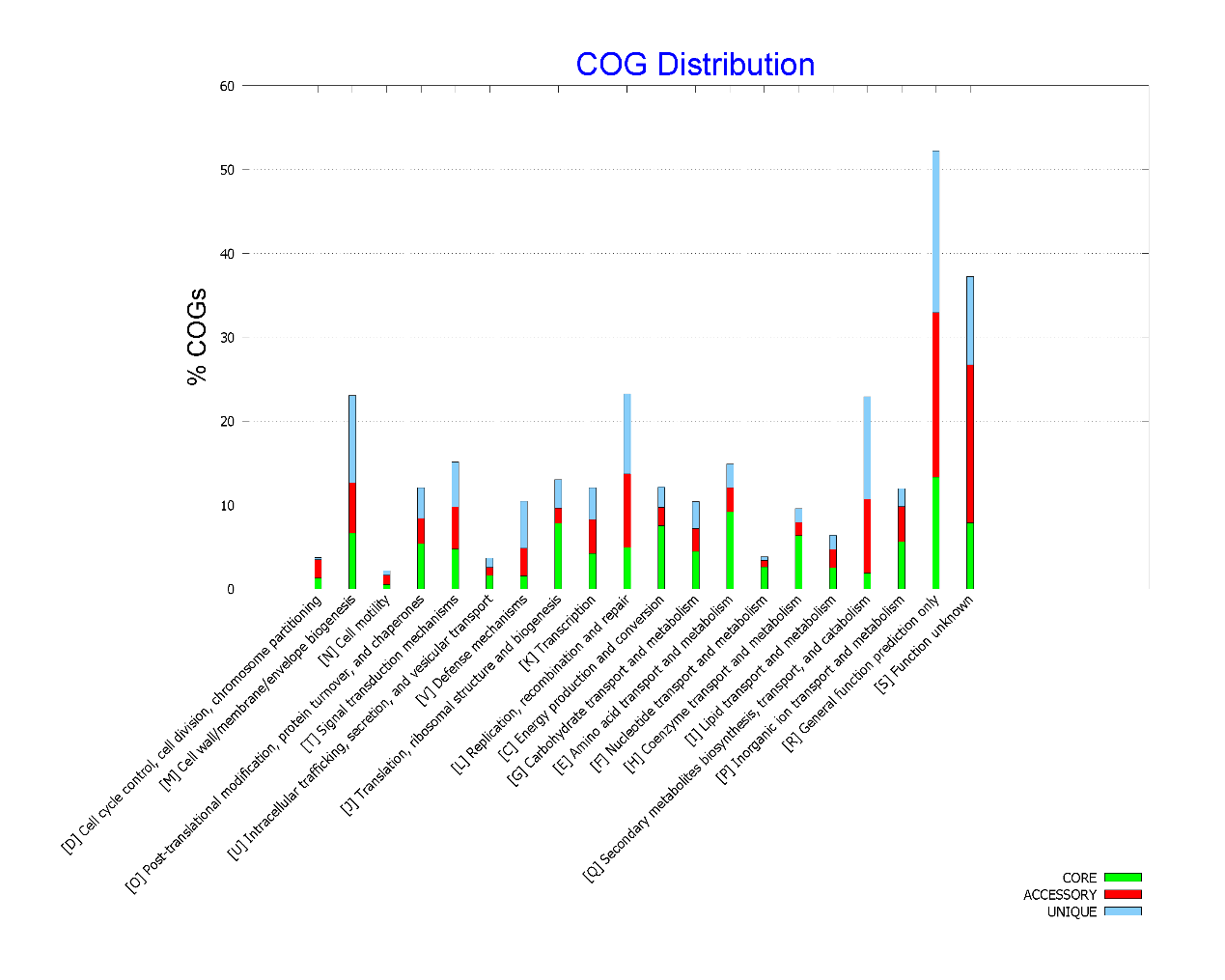
**

**Figure S1.** COG distribution of core, accessory and unique genes present in 24 analyzed *M. aeruginosa* genomes.

**
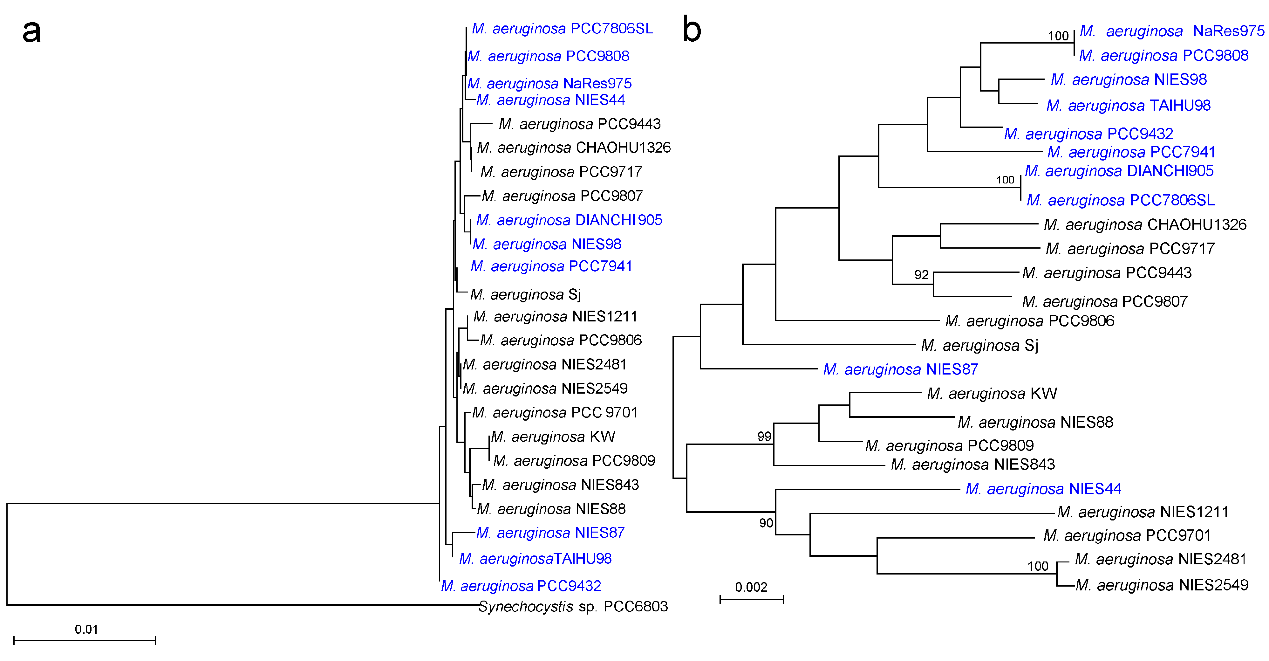
**

**Figure S2.** Phylogenetic analysis of *M. aeruginosa* strains. **(a)** Neighbour-joining phylogenetic tree based on the 16S rRNA gene sequences of 25 genomes. Twenty-five strains are used in this study plus *Synechocystis* sp. PCC 6803 as the outgroup. Bootstrap values above 90% are shown at the branch nodes (1,000 replicates). The scale bar represents 0.01 nucleotide substitutions per site. **(b)** Maximum likelihood phylogenetic tree based on multilocus sequence analysis of 31 concatenated conserved marker genes from 24 *M. aeruginosa* strains genomes using MEGA X. Bootstrap values above 90% are shown at the branch nodes (1,000 replicates). The scale bar represents 0.02 amino acid substitutions per site. Strains highlighted in blue represent that they do not possess REC-GGDEF domain containing DGC.


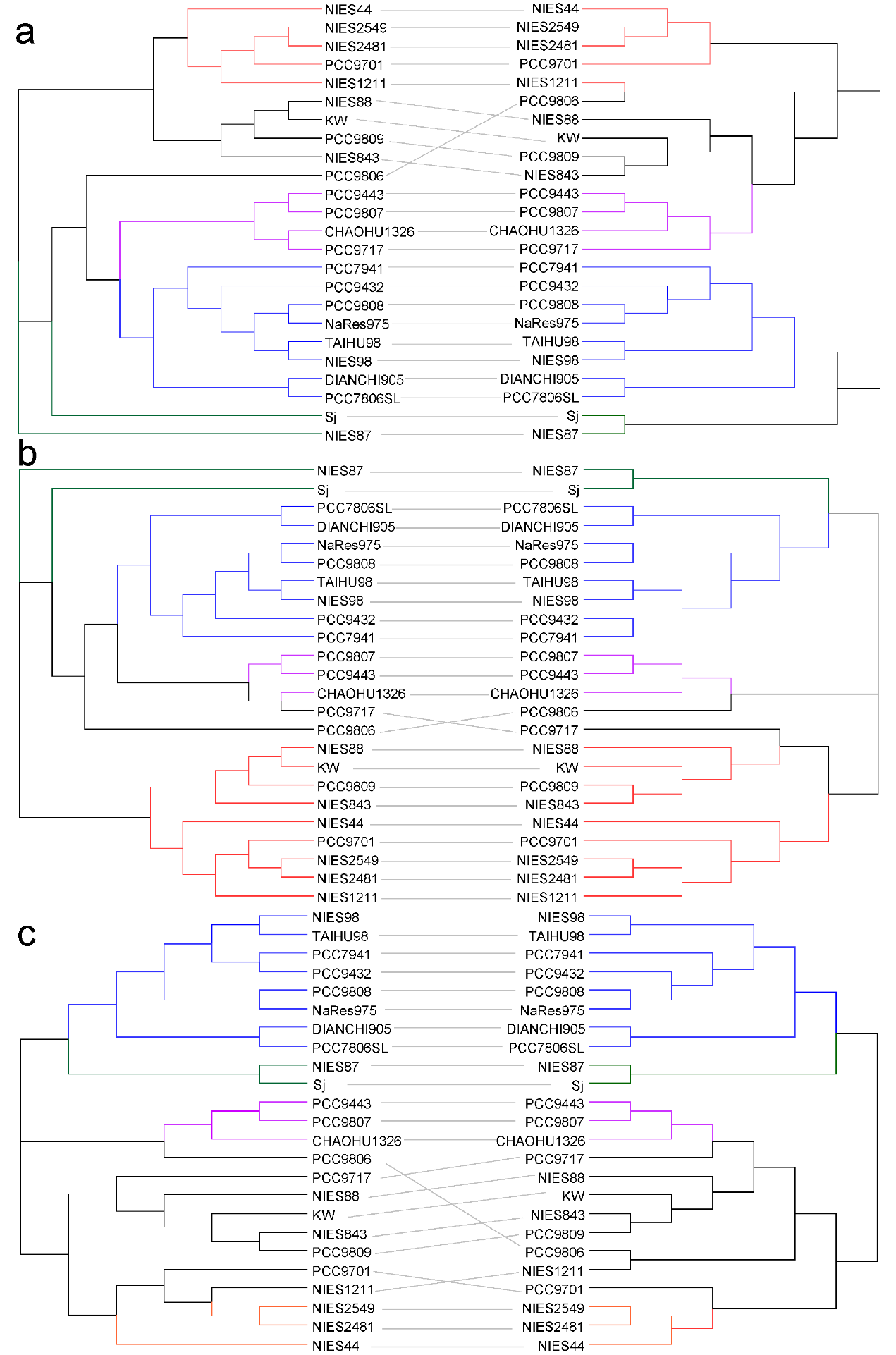


Figure S3. Tanglegram comparison of the phylogenetic trees. **(a)** The 31 marker genes tree (left) is compared with pan-genome phylogenomic tree (right). **(b)** The 31 marker genes tree (left) is compared with one generated using sensor genes tree (right). **(c)** The sensor genes tree (left) is compared with pan-genome phylogenomic tree (right).

**
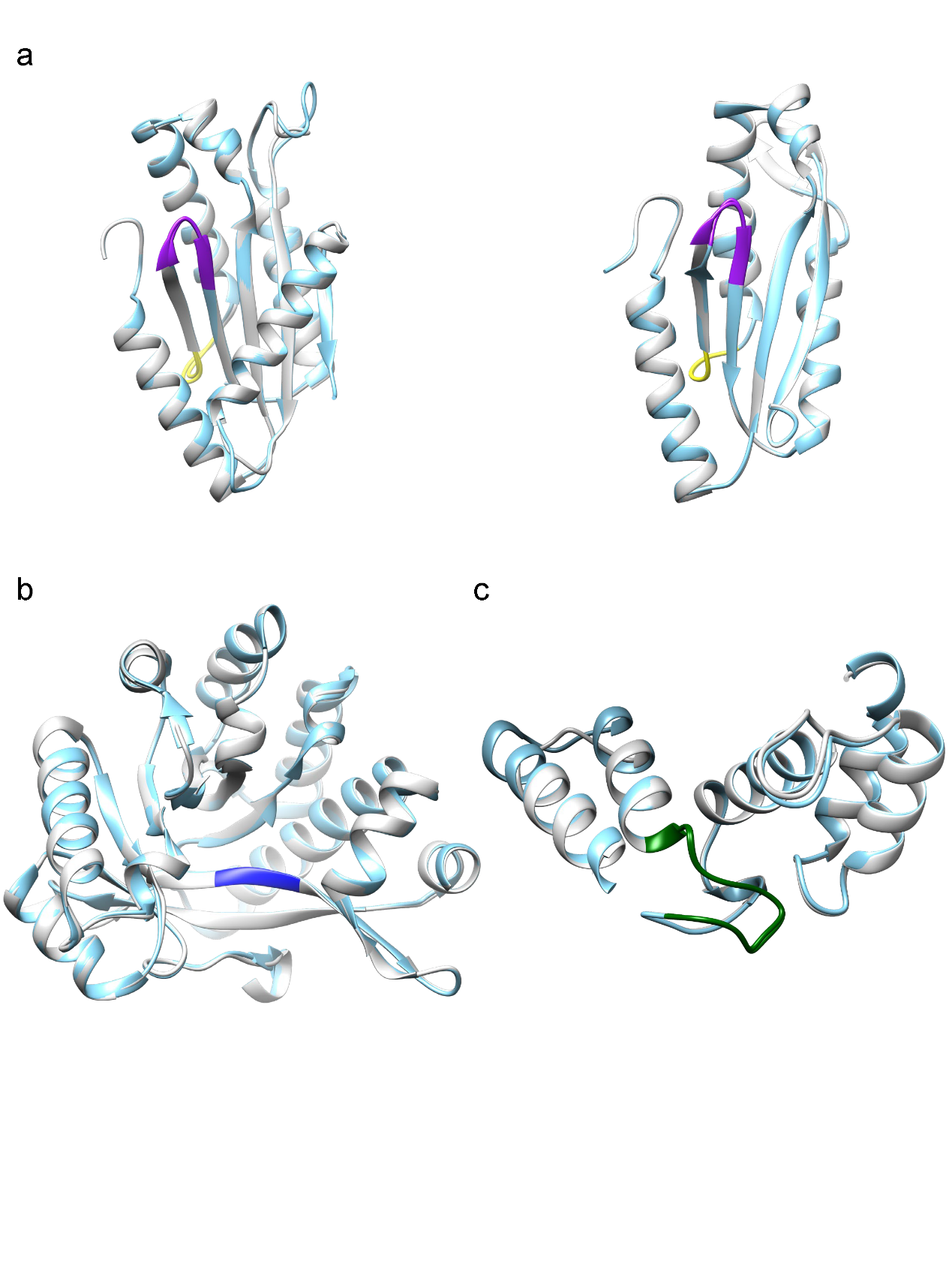
**

**Figure S4.** Structural features of GGDEF domain, and GGDEF-EAL domain from the *M. aeruginosa* CHAOHU1326 genome. The domain surface of CHAOHU 1326 are labeled in white, the templates are labeled in light blue. **(a)** GGDEF domain structures from the *M. aeruginosa* CHAOHU 1326 genome. Left, GGDEF domain taking crystal structure of WspR (PDB id: 3BRE) from *P. aeruginosa* as template. Right, GGDEF domain in hybrid protein, and RmcA (PDB id: 5M3C) from *P. aeruginosa* is used as template. **(b)** Domain structures of EAL domain in hybrid protein from the *M. aeruginosa* CHAOHU 1326 genome. RmcA (PDB id: 5M3C) from *P. aeruginosa* is used as template. The RXXD, GGEEF and EAL signature motif are labeled in yellow, purple and blue, respectively. **(c)** Structures of the HD-GYP domain of the *M. aeruginosa* CHAOHU1326. PA4781 (PDB id: 4R8Z) from *P. aeruginosa* was used as the template. The GYP loop signature motif is labeled in green.

**Table S1**. Genome features of *Microcystis aeruginosa* CHAOHU 1326 and NaRes975

| **Features** | **CHAOHU1326** | **NaRes975** |
| --- | --- | --- |
| **Total length (Mb)** | 5.27168 | 5.11753 |
| **Scaffold** | 607 | 413 |
| **Contig N50** | 19,902 | 29,122 |
| **Total genes** | 5,517 | 5,388 |
| **protein-coding genes** | 4,560 | 4,617 |
| **RNA-coding genes** | 59 | 47 |
| **pseudogenes** | 868 | 724 |

**Table S2.** Numbers of RNA genes found in all 24 analyzed *M. aeruginosa* genomes.

| Strains | tRNA | ncRNA | Complete rRNA | | | Partial rRNA | | |
| --- | --- | --- | --- | --- | --- | --- | --- | --- |
|  |  |  | 5S | 16S | 23S | 5S | 16S | 23S |
| CHAOHU 1326 | 46 | 4 | 3 | 1 | 1 | - | 4 | 10 |
| DIANCHI905 | 42 | 4 | 2 | 2 | 2 | - | 2 | - |
| KW | 42 | 4 | 2 | 2 | 2 | - | - | - |
| NaRes975 | 40 | 4 | 1 | 0 | 0 | - | 3 | 5 |
| NIES44 | 41 | 4 | 1 | 1 | 1 | - | - | - |
| NIES87 | 41 | 4 | 2 | 1 | 1 | - | - | - |
| NIES88 | 41 | 4 | 2 | 1 | 1 | - | - | - |
| NIES98 | 42 | 4 | 1 | 1 | 1 | - | - | 2 |
| NIES843 | 42 | 4 | 2 | 2 | 2 | - | - | - |
| NIES1211 | 41 | 4 | 1 | 1 | 1 | - | - | 4 |
| NIES2481 | 41 | 4 | 2 | 2 | 2 | - | - | - |
| NIES2549 | 41 | 4 | 2 | 2 | 2 | - | - | - |
| PCC7806SL | 42 | 4 | 2 | 2 | 2 | - | - | - |
| PCC7941 | 40 | 4 | 1 | 1 | 1 | - | - | - |
| PCC9432 | 40 | 4 | 1 | 1 | 1 | - | - | - |
| PCC9443 | 41 | 4 | 1 | 1 | 1 | - | - | - |
| PCC9701 | 41 | 4 | 1 | 1 | 1 | - | - | - |
| PCC9717 | 41 | 4 | 1 | 1 | 1 | - | - | - |
| PCC9806 | 41 | 4 | 1 | 1 | 1 | - | - | - |
| PCC9807 | 41 | 4 | 1 | 1 | 1 | - | - | - |
| PCC9808 | 40 | 4 | 1 | 1 | 1 | - | - | 1 |
| PCC9809 | 41 | 4 | 1 | 1 | 1 | - | - | 1 |
| Sj | 41 | 4 | - | 1 | 1 | 2 | - | - |
| TAIHU98 | 42 | 4 | 2 | 2 | 2 | - | - | - |

**Table S3.** Highly conserved GAF and PAS domain-containing protein accession numbers and domain architectures in *M. aeruginosa*.

| **Strains** | **S1**  **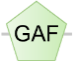** | **S2**  **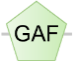** | **S3**  **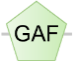** | **S4**  **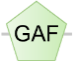** | **S5**  **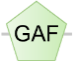** | **S6**  **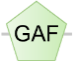** | **S7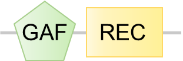** | **S8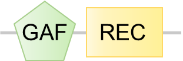** | **S9**  **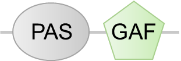** | **S10**  **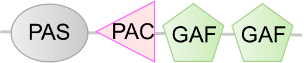** | **S11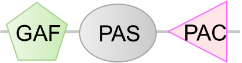** | **S12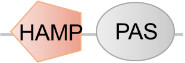** |
| --- | --- | --- | --- | --- | --- | --- | --- | --- | --- | --- | --- | --- |
| CHAOHU1326 | WP_072924073 | WP_072924730 | WP_052277404 | WP_072926573 | WP_072926580 | WP_052277305 | WP_072924626 | WP_072924963 | WP_052276811 | WP_072926064 | - | WP_002762449 |
| DIANCHI905 | WP_002745958 | WP_002747168 | WP_036399866 | WP_004157354 | WP_002747345 | WP_002748263 | WP_002741087 | WP_002747668 | WP_002744275 | WP_002741702 | WP_002740896 | WP_002742002 |
| KW | WP_079209549 | WP_079208831 | WP_079209700 | WP_079207421 | WP_079205485 | WP_079208235 | WP_079208383 | WP_079210006 | WP_079205924 | WP_079209813 | WP_079208453 | WP_079208968 |
| NaRes975 | WP_002794319 | - | WP_002791029 | WP_002793179 | WP_002754030 | WP_002793237 | WP_002794122 | WP_004162195 | WP_002794503 | WP_002791187 | WP_002794215 | WP_002791072 |
| NIES44 | WP_045357954 | WP_045357888 | WP_045360329 | WP_045360749 | WP_045359283 | WP_045357878 | WP_045359251 | WP_045361304 | WP_045362584 | WP_045356473 | WP_045357371 | WP_045361075 |
| NIES87 | WP_104395268 | WP_104396695 | WP_104397419 | - | WP_104396850 | WP_104397238 | WP_104395772 | WP_104396333 | WP_104396993 | WP_104398395 | WP_104397430 | WP_104395682 |
| NIES88 | WP_061431276 | WP_061433352 | WP_061433296 | WP_061431986 | WP_061431151 | WP_061430162 | WP_061433130 | WP_061432020 | WP_061432273 | WP_061430955 | WP_061432206 | WP_061432934 |
| NIES98 | WP_069475418 | - | WP_042790772 | WP_002776488 | WP_069474734 | WP_069474833 | WP_016515378 | WP_069473877 | WP_069475019 | WP_069474425 | WP_069475037 | WP_002735697 |
| NIES843 | WP_002798555 | - | WP_012265232 | WP_012266947 | WP_012263925 | WP_012265699 | WP_012265377 | WP_012267004 | WP_012264674 | WP_012267308 | WP_012265457 | WP_012266445 |
| NIES1211 | WP_106909322 | - | WP_110545822 | WP_110544865 | WP_008197420 | WP_008206790 | WP_008206225 | WP_110544472 | WP_008196709 | WP_110545383 | WP_008201845 | WP_008204843 |
| NIES2481 | WP_046661343 | WP_046662881 | WP_066029521 | WP_046663349 | WP_046663066 | WP_066029442 | WP_046661009 | WP_046661034 | - | WP_066030064 | WP_066029449 | WP_046662520 |
| NIES2549 | WP_046661343 | WP_046662881 | WP_046660921 | WP_046663349 | WP_046663066 | WP_046660624 | WP_046661009 | WP_046661034 | - | WP_046662941 | WP_046660687 | WP_046662520 |
| PCC7806SL | WP_002745958 | WP_002747168 | WP_002744306 | WP_004157354 | WP_002747345 | WP_002748263 | WP_002741087 | WP_002747668 | WP_002744275 | WP_084990071 | WP_002740896 | WP_002742002 |
| PCC7941 | WP_002773209 | WP_002778812 | WP_002773364 | WP_002776488 | WP_002774300 | WP_043997359 | WP_002753664 | WP_002776134 | WP_002777487 | WP_002753355 | WP_002778069 | WP_002773459 |
| PCC9432 | WP_002752739 | - | WP_002751457 | WP_002750080 | WP_002754030 | WP_043998257 | WP_002753664 | WP_002750596 | WP_004158869 | WP_002753355 | WP_002755152 | WP_002735697 |
| PCC9443 | WP_002768350 | WP_043996502 | WP_002765532 | WP_002772016 | WP_002767728 | WP_004159948 | WP_002766513 | WP_002772102 | WP_002769252 | WP_002770726 | WP_002768675 | WP_002765941 |
| PCC9701 | WP_004268082 | WP_004267903 | WP_002800894 | WP_002800080 | WP_002800292 | WP_043997878 | WP_002802777 | WP_002802735 | WP_002801722 | WP_004163700 | WP_002800792 | WP_002800602 |
| PCC9717 | WP_004159531 | WP_002757620 | WP_002759229 | WP_002764008 | WP_002760382 | WP_002756977 | WP_004266808 | WP_002757139 | WP_002758487 | WP_002759341 | WP_002758380 | WP_002762449 |
| PCC9806 | WP_002782883 | - | WP_002781396 | WP_002783547 | WP_002780554 | WP_002781783 | WP_002781723 | WP_002781098 | WP_002783718 | WP_002780998 | WP_002781582 | WP_002784436 |
| PCC9807 | WP_002789391 | WP_002785382 | WP_002787487 | WP_002786795 | WP_002786914 | WP_002789289 | WP_002787548 | WP_004161361 | WP_002785295 | WP_002785224 | WP_002789038 | WP_002790089 |
| PCC9808 | WP_002794319 | - | WP_002791029 | WP_002793179 | WP_002754030 | WP_002793237 | WP_002794122 | WP_004162195 | WP_002794503 | WP_002791187 | WP_002794215 | WP_002791072 |
| PCC9809 | WP_002798555 | WP_002798111 | WP_002798380 | WP_004162832 | WP_002797601 | WP_002799132 | WP_002798872 | WP_004162777 | WP_004163339 | WP_004162493 | WP_002796812 | WP_002798261 |
| Sj | WP_110578336 | WP_110578996 | WP_110578784 | WP_110579418 | WP_110578966 | WP_110577725 | WP_110578750 | WP_110577976 | WP_110579984 | WP_110578953 | WP_110579722 | WP_110579820 |
| TAIHU98 | WP_002732405 | - | WP_042790772 | WP_002740043 | WP_002737006 | WP_002739588 | WP_002733486 | WP_002737089 | WP_002732234 | WP_002733944 | WP_002733887 | WP_002735697 |

**Table S4.** Locations of genes related to c-di-GMP metabolism and regulation in *M. aeruginosa* NIES843

| **Label** | | **Accession numbers** | **Start** | **Stop** |
| --- | --- | --- | --- | --- |
| GAF | | WP_012263925.1 | 178494 | 180485 |
| PAS-GAF | | WP_012264674.1 | 1071281 | 1073587 |
| REC-GGDEF | | WP_012264732.1 | 1156256 | 1157188 |
| GAF | | WP_002798555.1 | 1284556 | 1285812 |
| GAF | | WP_012265232.1 | 1768099 | 1770336 |
| REC-GAF | | WP_012265377.1 | 1946692 | 1948701 |
| GAF-PAS-PAC | | WP_012265457.1 | 2034702 | 2040854 |
| GAF | | WP_012265699.1 | 2350509 | 2351267 |
| HAMP-PAS | | WP_012266445.1 | 3253137 | 3255134 |
| GGDEF-EAL | | WP_012266621.1 | 3478135 | 3479955 |
| GAF | | WP_012266947.1 | 3962283 | 3962702 |
| REC-GAF | | WP_012267004.1 | 4029476 | 4031671 |
| PAS-PAC-GAF-GAF | | WP_012267308.1 | 4408233 | 4410461 |
| DICT-HD-GYP | | WP_002796380.1 | 5235981 | 5237354 |
|  |  | |  |  |

**Table S5.** Positive selection for genes related to c-di-GMP metabolism and regulation in *M. aeruginosa*.

| Proteins | M1_lnL | M2_lnL | M2 vs M1^a^ | M7_lnL | M8_lnL | M7 vs M8^b^ |
| --- | --- | --- | --- | --- | --- | --- |
| S1 | -4064.04 | -4046.24 | 1.8521E-08 | -4064.53 | -4046.56 | 1.5663E-08 |
| S2 | -28747.66 | -28730.28 | 2.8311E-08 | -28744.94 | -28713.36 | 1.9274E-14 |
| S3 | -6626.50 | -6618.55 | 0.0004 | -6627.43 | -6618.55 | 0.0001 |
| S4 | -1336.83 | -1319.34 | 2.5403E-08 | -1336.99 | -1319.03 | 1.5849E-08 |
| S5 | -5029.20 | -5023.57 | 0.0036 | -5029.32 | -5023.80 | 0.0040 |
| S6 | -2510.85 | -2499.13 | 8.0551E-06 | -2510.97 | -2499.09 | 6.8984E-06 |
| S7 | -5501.19 | -5471.40 | 1.1530E-13 | -5506.38 | -5471.64 | 8.1876E-16 |
| S8 | -6843.90 | -6823.40 | 1.2459E-09 | -6847.05 | -6822.92 | 3.3216E-11 |
| S9 | -5650.00 | -5635.02 | 3.1276E-07 | -5650.19 | -5634.92 | 2.3344E-07 |
| S10 | -7094.68 | -7081.52 | 1.9173E-06 | -7100.53 | -7081.61 | 6.0616E-09 |
| S11 | -20133.7 | -20052.6 | 5.9414E-36 | -20135.9 | -20053.3 | 1.3364E-36 |
| S12 | -5307.77 | -5305.17 | 0.0742 | -5307.91 | -5305.18 | 0.0649 |
| DGC | -3073.51 | -3065.78 | 0.0004 | -3074.01 | -3065.79 | 0.0003 |
| PDE | -3246.66 | -3244.31 | 0.0959 | -3250.02 | -3244.34 | 0.0034 |
| Hybrid | -6348.28 | -6345.46 | 0.0594 | -6348.42 | -6345.52 | 0.0553 |

^a^ and ^b^: likelihood-ratio testing M2 vs M1 and M8 vs M7, respectively.

Table S6 was shown at Additional file 2.

**Table S7.** QMEAN Z-score of the predicted structures of EAL, GGDEF, HD-GYP and PilZ domain containing proteins.

| **Strains** | **Proteins** | **Accession numbers** | **QMEAN Z-score** |
| --- | --- | --- | --- |
| NIES843 | DGC | WP_012264732.1 | -1.65 |
| NIES843 | Hybrid protein | WP_012266621.1 | -0.93 |
| NIES843 | PDE(HD-GYP) | WP_002796380.1 | -1.44 |
| NIES843 | PilZ | BAG05170.1 | -3.04 |
| CHAOHU 1326 | DGC | WP_052276147.1 | -1.37 |
| CHAOHU 1326 | Hybrid protein | WP_052277914.1 | -1.04 |
| CHAOHU 1326 | PDE(HD-GYP) | WP_052275339.1 | -1.57 |
| CHAOHU 1326 | CelA | WP_052275940.1 | -4.0 |

**Table S8.** Identity of DGC sequences from *M. aeruginosa* genomes compared to WspR from *P. aeruginosa*.

| **Strains** | **Accession numbers** | **Identity (%)** |
| --- | --- | --- |
| CHAOHU 1326 | WP_052276147.1 | 35.11 |
| KW | WP_079210059.1 | 35.11 |
| NIES88 | WP_061433230.1 | 35.33 |
| NIES843 | WP_012264732.1 | 35.11 |
| NIES1211 | WP_039900524.1 | 35.56 |
| NIES2481 | WP_046660716.1 | 37.22 |
| NIES2549 | WP_046660716.1 | 37.22 |
| PCC9443 | WP_043996837.1 | 34.17 |
| PCC9701 | WP_002801860.1 | 37.3 |
| PCC9717 | WP_043999403.1 | 36.22 |
| PCC9806 | WP_002783698.1 | 34.8 |
| PCC9807 | WP_002787322.1 | 35.65 |
| PCC9809 | WP_043999403.1 | 36.22 |
| Sj | WP_110579156.1 | 34.17 |

**Table S9**. Identity of GGDEF-EAL domain sequences from *M. aeruginosa* genomes compared to that from *P. aeruginosa*.

| **Strains** | **Accession numbers** | **Identity (%)** |
| --- | --- | --- |
| CHAOHU 1326 | WP_052277914.1 | 37.83 |
| DIANCHI905 | WP_002743531.1 | 36.17 |
| KW | WP_079210289.1 | 37.35 |
| NaRes975 | WP_044034220.1 | 36.23 |
| NIES87 | WP_104397223.1 | 36.41 |
| NIES88 | WP_061431785.1 | 37.83 |
| NIES98 | WP_002739484.1 | 35.92 |
| NIES843 | WP_012266621.1 | 37.21 |
| NIES1211 | WP_110544382.1 | 37.12 |
| NIES2481 | WP_066029445.1 | 36.04 |
| NIES2549 | WP_046660636.1 | 36.28 |
| PCC7806SL | WP_002743531.1 | 36.17 |
| PCC7941 | WP_043997363.1 | 35.92 |
| PCC9432 | WP_002750015.1 | 36.17 |
| PCC9443 | WP_002768060.1 | 37.26 |
| PCC9701 | WP_004163835.1 | 36.17 |
| PCC9717 | WP_002761714.1 | 36.66 |
| PCC9806 | WP_002783280.1 | 36.5 |
| PCC9807 | WP_004161732.1 | 37.59 |
| PCC9808 | WP_044034220.1 | 36.23 |
| PCC9809 | WP_002797049.1 | 37.35 |
| Sj | WP_110577728.1 | 37.47 |
| TAIHU98 | WP_002739484.1 | 35.92 |

**Table S10**. Identity of HD-GYP containing PDE sequences from *M. aeruginosa* genomes compared to that from *P. aeruginosa*.

| **Strains** | **Accession numbers** | **Identity (%)** |
| --- | --- | --- |
| CHAOHU 1326 | WP_052275339.1 | 33.58 |
| DIANCHI905 | WP_002746813.1 | 34.33 |
| KW | WP_002796380.1 | 34.33 |
| NaRes975 | WP_002752229.1 | 34.33 |
| NIES44 | WP_045358386.1 | 34.33 |
| NIES87 | WP_104396273.1 | 34.33 |
| NIES88 | WP_061432432.1 | 34.33 |
| NIES98 | ODV36694.1 | 34.33 |
| NIES843 | WP_002796380.1 | 34.33 |
| NIES1211 | WP_039900517.1 | 34.33 |
| NIES2481 | WP_066029831.1 | 34.33 |
| NIES2549 | WP_046662116.1 | 34.33 |
| PCC7806SL | WP_002746813.1 | 34.33 |
| PCC7941 | WP_002752229.1 | 34.33 |
| PCC9432 | WP_002752229.1 | 34.33 |
| PCC9443 | WP_002765696.1 | 33.58 |
| PCC9701 | WP_002803155.1 | 34.33 |
| PCC9717 | WP_002762031.1 | 34.33 |
| PCC9806 | WP_002780038.1 | 34.33 |
| PCC9807 | WP_002785975.1 | 33.58 |
| PCC9808 | WP_002752229.1 | 34.33 |
| PCC9809 | WP_002796380.1 | 34.33 |
| Sj | WP_110579081.1 | 33.58 |
| TAIHU98 | WP_002733640.1 | 34.33 |

**Table S11.** Accession numbers of the predicted PilZ containing proteins found in 24 analyzed *M. aeruginosa* genomes.

| **Strains** | **Accession numbers** | **Product** |
| --- | --- | --- |
| CHAOHU 1326 | WP_052275940.1 | CelA |
| DIANCHI905 | WP_002743951.1 | PilZ |
| KW | WP_079209287.1 | CelA |
| NaRes975 | WP_002791984.1 | CelA |
| NIES44 | WP_045358830.1 | CelA |
| NIES87 | WP_104396371.1 | CelA |
| NIES88 | WP_072319631.1 | PilZ |
| NIES98 | WP_069475389.1 | CelA |
| NIES843 | BAG05170.1 | PilZ |
| NIES1211 | WP_110545972.1 | CelA |
| NIES2481 | WP_046661748.1 | CelA |
| NIES2549 | WP_046661748.1 | CelA |
| PCC7806SL | WP_084989905.1 | CelA |
| PCC7941 | WP_002776694.1 | CelA |
| PCC9432 | WP_002755033.1 | CelA |
| PCC9443 | WP_002769952.1 | CelA |
| PCC9701 | WP_004268239.1 | CelA |
| PCC9717 | WP_002759615.1 | CelA |
| PCC9806 | WP_002780702.1 | CelA |
| PCC9807 | WP_002787005.1 | CelA |
| PCC9808 | WP_002791984.1 | CelA |
| PCC9809 | CCI22636.1 | PilZ |
| Sj | WP_110578231.1 | CelA |
| TAIHU98 | WP_042791107.1 | CelA |
